# Controlling Selectivity in Electrochemical Conversion of Organic Mixtures through Dynamic Control of Electrode Microenvironments

**DOI:** 10.1021/jacs.5c12445

**Published:** 2025-10-04

**Authors:** Ricardo Mathison, Elina Rani, Amelia M. Rose, Fjona Prendi, Casey K. Bloomquist, Miguel A. Modestino

**Affiliations:** Department of Chemical and Biomolecular Engineering, 5894 New York University, Brooklyn, New York 11201, United States

## Abstract

Organic electrosynthesis using renewable electricity offers a sustainable approach to chemical manufacturing. Among its promising applications, the selective transformation of complex organic mixtures presents a valuable opportunity to eliminate costly separation processes and directly convert heterogeneous feedstocks to valuable products. However, controlling selectivity in reaction mixtures remains challenging due to competing reaction pathways and varying reactivities among substrates. Here, we demonstrate how selectivity in mixed organic electrosynthesis can be systematically controlled through a balance of reaction kinetics and mass transport limitations. Using acrylonitrile and crotononitrile mixtures as a model substrate mixture, we established quantitative relationships between substrate compositions, current densities, and product distributions that reveal distinct kinetically limited and mass transport-limited reaction regimes that control selectivity. We further demonstrated how pulsed electrolysis can be used to strategically control these reaction regimes to drive selectivity toward specific products. These insights create opportunities for developing adaptive and dynamic chemical manufacturing processes capable of handling complex feedstocks.

## Graphical abstract

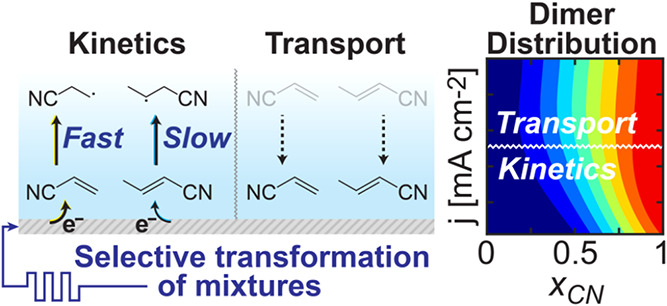

## Introduction

Over the past few decades, organic electrosynthesis has experienced a remarkable resurgence through the development of standardized equipment, which has accelerated the rate of reaction discovery and development.^1^ This synthetic approach had been historically underutilized by organic chemists due to the lack of accessible tools for implementation despite its potential to enable previously inaccessible transformations.^2–4^ Furthermore, organic electrosynthesis offers a promising strategy for decarbonizing the chemical industry by using renewable electricity to drive chemical reactions under milder conditions than conventional thermal methods, reducing fossil fuel dependence, energy consumption, and waste generation in large-scale manufacturing.^5–8^ However, the practical implementation of organic electrosynthesis at the scale has been limited by several electrochemical reaction engineering challenges. While aqueous electrolytes offer advantages such as high ionic conductivity, safety, and cost-effectiveness for large-scale implementation, they present unique challenges for organic substrates. Key challenges include low conversion rates due to poor solubility of organic reactants in aqueous media,^9,10^ decreased conductivity in the presence of organic phases,^11^ and limited electrochemical stability of aqueous electrolytes.^12^ Furthermore, the presence of multiple competing reaction pathways leads to unwanted byproducts, a challenge in synthetic chemistry that is particularly exacerbated when dealing with multicomponent organic mixtures or molecules with multiple reaction centers.^13–15^ Electrochemistry offers unique advantages through precise potential control, enabling selective transformation of complex organic mixtures that could eliminate costly separation processes while enabling direct conversion to valuable products. Achieving selectivity in such reaction mixtures requires precise control over both reaction kinetics and mass transport to direct electron transfer toward specific pathways when multiple reactive species are present. Organic mixtures exemplify this selectivity challenge, as their heterogeneous nature and varied composition typically demand energy-intensive separation processes before conventional processing.^16–18^ For instance, lignin is a complex aromatic polymer from biomass that presents significant challenges in electrochemical conversion due to its intricate three-dimensional network with diverse chemical linkages that limits electrode access to reaction sites,^19^ while also leading to low selectivities due to competing hydrogen and oxygen evolution reactions.^20^ To address these limitations, researchers have developed strategies that manipulate mass transport dynamics, including slurry reactor configurations,^21,22^ redox-mediated reactions,^23,24^ and ionic liquid-based approaches.^25^ Other strategies have emerged to control selectivity in organic mixtures such as electrode surface modifications for selective substrate discrimination,^26^ and electron–proton transfer mediators that discriminate between functional groups.^27^ However, there is a lack of a general understanding of how the delicate balance between reaction kinetics and mass transport phenomena governs selective transformations in complex organic mixtures.

Dynamic electrochemical techniques that modulate the interplay between transport and kinetics have shown promise for controlling the reaction selectivity. Rapid alternating polarity electrolysis has emerged as a powerful approach for achieving selective reduction of specific functional groups like phthalimides in the presence of other reducible groups, demonstrating control through frequency-dependent electron transfer kinetics.^28^ More broadly, pulsed electrochemical methods have gained significant traction as a mainstream approach to increase electrolyzer durability and improve product selectivity, particularly for CO_2_ reduction reactions and other electrochemical processes involving competing reactions or electrode deactivation.^29–32^ These dynamic approaches manipulate the near-electrode microenvironment, strategically creating conditions where kinetic differences between competing substrates can be exploited or mitigated. Despite these advances, achieving consistent selectivity across diverse reaction types and substrates remains a significant challenge. Understanding how potential modulation controls selectivity toward specific reaction pathways would enable the development of highly specific electrosynthetic processes that maximize target molecule production.

To address this knowledge gap, we explored the electrohydrodimerization of mixtures of acrylonitrile (AN) and crotononitrile (CN) as a model organic reactive mixture. Our work systematically examine how the balance between inherent kinetic differences and mass transport limitations governs product selectivity, establishing a mechanistic framework for controlling reactions in mixed-substrate systems. This framework centers on understanding how local reactant concentrations at the electrode surface, governed by the interplay of diffusion rates, bulk concentration, and consumption kinetics, determine which reaction pathways dominate. The choice of this model reaction was inspired by the electrohydrodimerization of AN to adiponitrile (ADN), an important Nylon-6,6 precursor, which achieved practical viability through Baizer and Danly’s pioneering work^33,34^ and stands as one of the most successful industrial organic electrosynthesis process.^35,36^ Recent work from our group has advanced the understanding of how mass transport phenomena influence reaction outcomes in this reaction, and how to control them via electrolyte design,^37,38^ convection methods,^39^ pulsing strategies,^40^ and near-electrode microenvironment control.^41^ However, fundamental questions remain about how the substrate molecular structure influences electrohydromerization rates and how dynamic potential modulation controls dominant reaction pathways in mixtures of vinyl nitriles.

In this study, we leveraged an accelerated reaction engineering approach to investigate selective electrosynthesis in organic mixtures. To efficiently explore this complex parameter space, we employed a semiautonomous research methodology combining high-throughput experimentation with data-driven surrogate modeling. Through systematic experimentation, we established quantitative relationships between AN/CN substrate compositions and current densities that govern product selectivities, revealing regimes where reactions are kinetically limited versus mass transport limited. Electron Paramagnetic Resonance (EPR) spectroscopy allowed us to quantify the relative radical intermediate generation that determines the varying production rates across substrate compositions. Our findings reveal how molecular differences between substrates create reactivity differences that manifest in both radical formation and coupling rates, directly influencing the product selectivity. We developed performance metrics to quantify dimer selectivity and substrate incorporation, providing a more nuanced understanding of how reaction parameters influence the product distribution in complex mixtures. Additionally, we demonstrated how pulsed electrolysis creates conditions in which both faster- and slower-reacting molecules can be present at the electrode surface in balanced amounts, enabling precise control over electrode surface species to enhance the formation of specific dimers. These experimental findings significantly advance our understanding of the interplay of reaction parameters that control electrode–electrolyte microenvironments in mixed organic electrosynthesis, offering applicable insights for selective electrochemical transformations of organic mixtures.

## Results and Discussion

### Product Distribution and Mechanistic Insights in Mixed-Substrate Electrosynthesis

The electroreduction of vinyl nitriles has been previously studied, with AN electrodimerization to ADN representing the most significant industrial example due to its role in Nylon production. Early insights from Baizer’s studies of α,β-unsaturated acid derivatives demonstrated β-position coupling to form dimers under reductive conditions and α-position hydrogenation across various substrates.^42^ The electroreduction of AN follows two primary reaction pathways: dimerization to form ADN and hydrogenation to produce propionitrile (PN). Selectivity toward ADN is maximized when high reactant concentrations are maintained at the electrode surface, which occurs under low current densities and high AN bulk concentrations.^37^ As the current density increases and AN is consumed at faster rates, the reaction becomes mass transport limited. This condition requires a higher bulk AN concentration to suppress hydrogenation pathways and maintain high ADN selectivity, highlighting the critical role of mass transport control in determining reaction selectivity.^41^

Selectivity control becomes even more complex when a second substrate is present. In our study, we use CN, a methyl-substituted acrylonitrile, as it provides a structurally similar yet distinct substrate to probe selectivity effects in multicomponent systems. Mixed AN/CN substrates create a network of competing reaction pathways, including both dimerization and hydrogenation, as shown in Figure 1A. Both substrates undergo reduction and protonation at the electrode surface. The resulting radical intermediates can then follow different reaction paths. Through radical coupling, three possible dimers can form: adiponitrile (ADN–AN dimer), 3-methyladiponitrile (ACDN–AN-CN mixed dimer), and 3,4-dimethyladiponitrile (CDN–CN dimer). Alternatively, the intermediate radicals can undergo further reduction and protonation, yielding the hydrogenation products: propionitrile (PN-saturated AN) and butyronitrile (BN-saturated CN).

**Figure 1:**
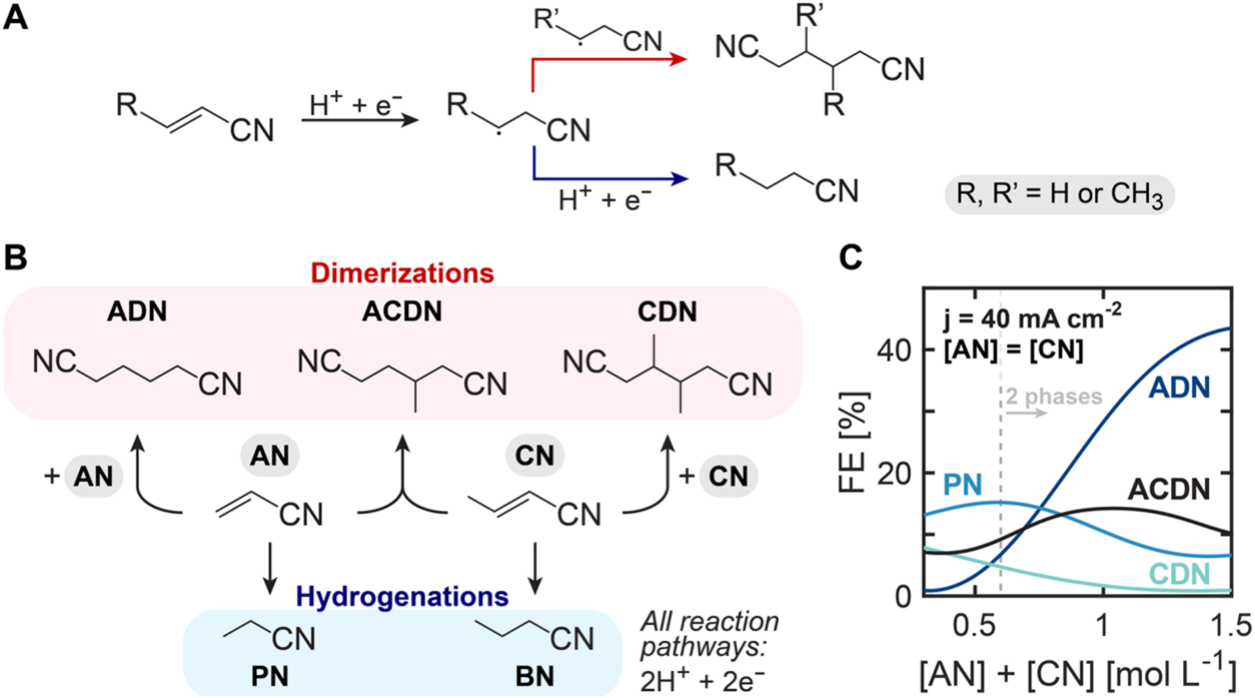
Electrochemical reduction pathways of acrylonitrile and crotononitrile electrolyte mixtures. (A) Proposed mechanism of the reactions present in the electroreduction of vinyl nitriles. (B) Dimerization products are ADN (AN hydrodimer), ACDN (AN-CN mixed dimer), and CDN (CN hydrodimer); and hydrogenation products are PN (saturated AN) and BN (saturated CN). (C) Effect of AN and CN (substrates) combined concentration on selectivity toward AN-hydrogenation product (PN) and dimerization products (ADN, ACDN, CDN) for electroreduction on a Cd rod at −40 mA cm^–2^. There was negligible production of the CN hydrogenation product (BN). Reactions were carried out in undivided parallel reactors. Electrolytes contained 0.5 mol L^–1^ Na_3_PO_4_, 0.03 mol L^–1^ EDTA, and 0.02 mol L^–1^ TBA hydroxide. The results are derived from a Gaussian Process Regression (GPR) surrogate model trained on 26 experimental observations dispersed through the 1D space.

Product distributions in the mixed AN/CN reaction system are governed by the interplay between mass transport limitations and reaction kinetics. In this system, both the substrate availability at the electrode surface and the relative rates of competing reactions determine the ultimate selectivity. As an initial exploration of this relationship, we examined equimolar mixtures of AN and CN ([AN] *=* [CN]) at different total substrate concentrations ([Substrate] = [AN] + [CN]) with the resulting product selectivities shown in Figure 1B.

Hydrogenation of AN to PN is the favored organic reaction at low substrate concentrations, where mass transport limitations dominate. Notably, we observed only AN hydrogenation to produce PN, while CN hydrogenation to BN was observed at negligible rates for all experimental conditions. Dimerization reactions emerge as the favored products as [Substrate] increases and mass transport limitations decrease. However, the dimers are not produced equally: ADN is strongly preferred over the mixed dimer (ACDN), and CDN production is effectively suppressed despite the equal bulk concentrations of AN and CN.

These observations can be explained based on a balance between mass transport and kinetic limitations. We demonstrated in previous mechanistic studies that dimerization proceeds through free radical coupling in solution, while hydrogenation likely occurs primarily as a surface reaction.^41^ The methyl substituent in CN likely influences its reactivity through two effects: First, it introduces steric hindrance that may interfere with proton transfer at the electrode surface. Second, it stabilizes the radical intermediate, maintaining the reduced substrate predominantly in the solution phase, where radical coupling is favored over surface hydrogenation. These effects collectively shift CN reactivity toward dimerization pathways rather than toward hydrogenation. Furthermore, the absence of BN and the preferential production of ADN and ACDN over CDN suggests that PN radical (PN^•^) formation occurs faster than BN radicals (BN^•^) (Figure 2A), leading to higher local concentrations of PN^•^ and increasing the probability of PN^•^–PN^•^ coupling over BN^•^–BN^•^ coupling. In addition, BN^•^ predominantly forms the mixed dimer ACDN through coupling with the more abundant PN^•^ rather than self-coupling to form CDN. The lower formation of BN^•^ relative to PN^•^ could also be attributed to the limited CN solubility in water (0.3 mol L^–1^), which significantly limits the flux of CN molecules to the electrode surface; when the CN concentration exceeded this solubility limit, a second organic phase formed on top of the aqueous electrolyte. This solubility limitation further contributes to the observed product selectivity by creating an imbalance in the availability of the two substrates at the electrode surface despite their equal bulk concentrations.

**Figure 2:**
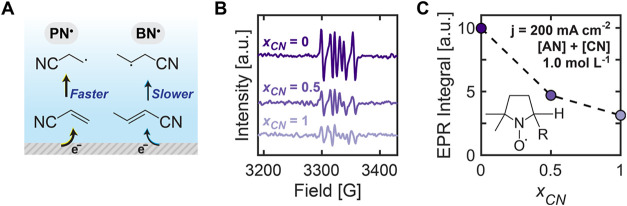
Effect of the substrate concentration on the product distributions for equimolar AN and CN. (A) Schematic representation of free radical generation on the near-electrode microenvironment for equimolar AN and CN mixtures. (B) EPR spectra instantly obtained after 10 min of electrolysis at constant −200 mA cm^–2^ on Cd foil with varying substrate composition given by CN relative molar fraction (*x*_CN_). The electrolyte contained 1.0 mol L^–1^ of substrate and 0.37 M DMPO (424 mg). (C) Relative concentration of free radicals given by the EPR double integral. All electrolytes contained 0.5 mol L^–1^ of Na_3_PO_4_, 0.03 mol L^–1^ of EDTA, and 0.02 mol L^–1^ of TBA hydroxide.

To explain the observed preference for AN-derived products, we experimentally quantified the relative rates of radical formation using EPR spectroscopy and radical trapping to directly detect these species in solution. Due to the short-lived nature of the produced alkyl radicals, the spin trap 5,5-dimethyl-1-pyrroline *N*-oxide (DMPO) was added to form stable DMPO-radical adducts detectable by EPR. We then performed experiments using AN/CN mixtures with different relative molar fractions of CN, *x*_CN_, defined as

$$x_{\mathrm{CN}}=\frac{n_{\mathrm{CN}}}{n_{\mathrm{AN}}+n_{\mathrm{CN}}}=\frac{\left[\mathrm{CN}\right]}{\left[\mathrm{AN}\right]+\left[\mathrm{CN}\right]}$$

where *n*_CN_ and *n*_AN_ are the moles of CN and AN, respectively. Electroreduction of AN/CN at *x*_CN_ values of 0, 0.5, and 1 was performed on Cd foil under chronopotentiometry conditions at −200 mA cm^–2^ for 10 min with excess spin trap to ensure a higher concentration compared to electrochemically generated radicals. The EPR spectra collected upon reaction completion for all *x*_CN_ values confirmed the presence of alkyl radicals (Figure 2B). The six observed peaks showed agreement with previously reported DMPO-carbon-centered radical adducts^43,44^ and with the simulated spectra of ^•^DMPO–CH_2_CH_2_CN.^41^ A qualitative comparison of relative radical concentrations was achieved by double integrating the EPR signals, with these comparative values shown in Figure 2C. The results clearly demonstrate that systems with pure AN (*x*_CN_ = 0) produce substantially more radicals than those with pure CN (*x*_CN_ = 1). Mixed-substrate reaction systems (*x*_CN_ = 0.5) show intermediate radical concentrations. This confirms that AN reduction to form PN^•^ is favored over CN reduction to BN^•^, directly explaining why ADN becomes the predominant product when the reaction is not mass transport limited.

### Balancing Reactivity and Transport in Mixed-Substrate Electrosynthesis

Building on our findings regarding kinetic limitations and mass transport effects, we then explored how tuning multiple reaction parameters simultaneously influences the product distribution in electrochemical olefin dimerization with mixed substrates. In the previous section, we established that product distribution is determined by both species’ reactivity and species concentrations in the local microenvironment near the electrode surface. While our initial study focused on total substrate concentration and current density with a fixed equimolar ratio, we now demonstrate that all three variables can be systematically adjusted to balance kinetics and transport and thus control selectivity. Bulk substrate concentration plays a crucial role in controlling mass transport by influencing the diffusion of species from the bulk solution to the near-electrode surface. Molar ratios between reactants determine the relative concentrations of competing substrates at the electrode surface, directly influencing product selectivity. Current density governs the overall reaction rate and the consumption of reactants, with higher current densities creating steeper concentration gradients that intensify the mass transport limitations near the electrode. This comprehensive approach reveals how seemingly competitive reactions can be directed toward specific products through the strategic parameter selection.

Investigating these interrelated parameters using conventional approaches would be prohibitively time-consuming. Therefore, we developed a semiautonomous accelerated electrochemical research methodology that combines high-throughput experimentation (HTE) with machine learning techniques to build data-driven surrogate models (Figure 3).^45^ We selected experimental conditions using Hammersley sampling, a pseudorandom sampling technique, to comprehensively and efficiently sample the parameter space.^46^ Next, we performed hundreds of electrosynthesis experiments at the selected conditions using our HTE workflow with a parallel electrochemical reactor^47^ coupled with a liquid-handling robot,^48^ enabling electrolyte preparation and parallel liquid–liquid extractions of organic molecules in the liquid phase for chemical analysis. The collected experimental data was used to construct surrogate models using Gaussian process regression (GPR) that relate reaction parameters (substrate concentration, molar fraction, and current density) to output variables such as selectivity and production rate. These data-driven models allowed us to examine trends in parameter-product relationships across the entire experimental domain. Model validation was performed by evaluating the standard deviation provided by GPR, with additional experimental verification conducted where necessary. Detailed information regarding model construction, performance testing, and uncertainty analysis can be found in the Supporting Information (Figures S1–S3). Following this HTE approach, we performed a more detailed analysis of the reaction under a few relevant set of conditions using a gastight H-cell batch reactor, which allowed us to collect and quantify gas products and characterize the complete product distribution from the reactions (Figures S4–S7). These H-cell experiments also enabled comparison of unreacted substrates before and after reaction to assess their relative conversions.

**Figure 3:**
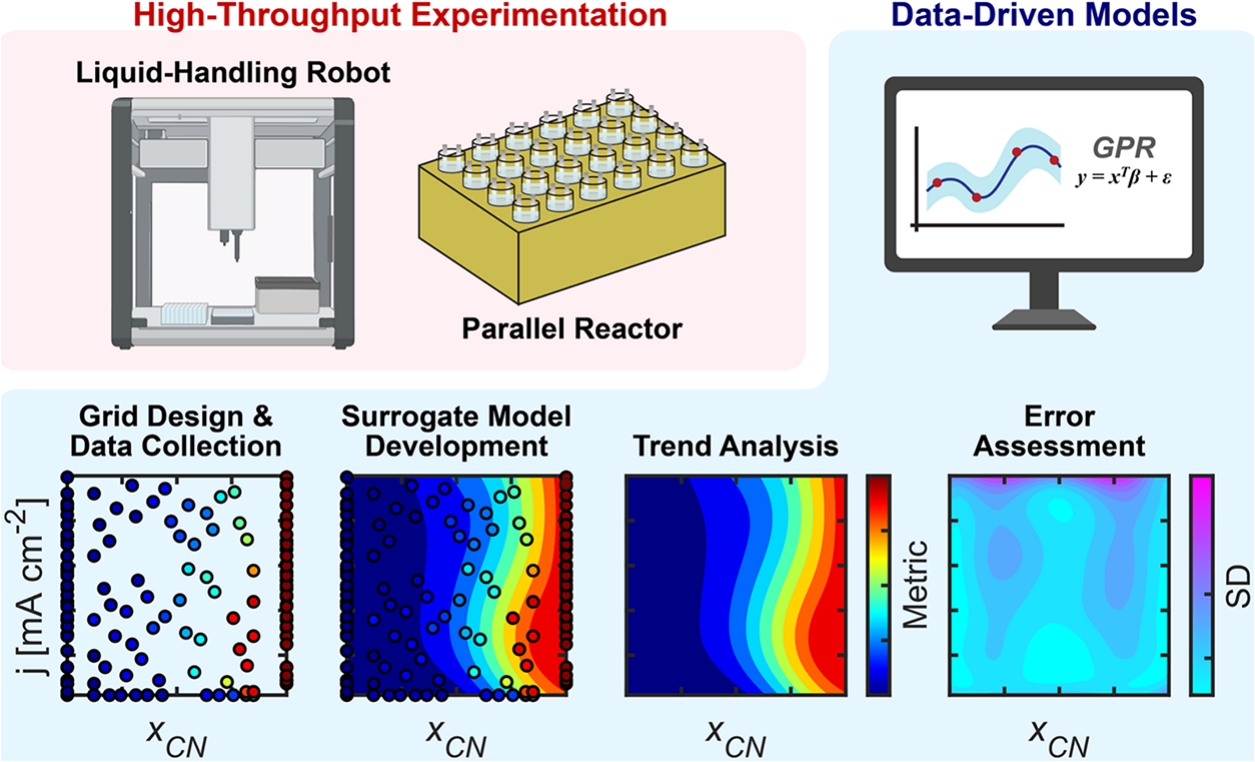
Semiautonomous research methodology combining high-throughput experimentation with data-driven surrogate modeling. The HTE combines a parallel electrochemical reactor with a liquid-handling robot for electrolyte preparation and parallel liquid–liquid extractions of organic molecules in the liquid phase for chemical analysis. Data-driven models were built following these steps: experimental design and data collection, combining Hammersley sampling for condition selection with HTE electrochemical data collection; surrogate model development, using GPR to establish relationships between reaction conditions and performance metrics; trend analysis, examining parameter-product relationships through heat-map visualizations; and error assessment, evaluating the GPR model’s standard deviation (SD) to identify regions with high uncertainty.

### Product Distribution under Different Mass Transport Regimes

The interplay between substrate concentration ([Substrate]), molar fraction of CN (*x*_CN_), and current density (*j*) on the product distribution is illustrated in Figure 4. For clarity, we present results for two substrate concentration regimes: a mass transport-limited system ([Substrate]_low_ = 0.6 mol L^–1^) and a system with reduced mass transport limitations ([Substrate]_high_ = 1.0 mol L^–1^). Our analysis focuses on the reactivity of pure components (*x*_CN_ = 0 or 1) before examining mixed-substrate compositions. For pure AN electrolytes (*x*_CN_ = 0) under mass transport-limited conditions ([Substrate]_low_, Figure 4A), we observed ADN production predominantly at low current densities. As the current density increased, mass transport limitations intensified, resulting in decreased FE_ADN_ and increased FE_PN_. This shift occurs because higher current densities accelerate reactant consumption at the electrode surface, creating steeper concentration gradients that favor the formation of the hydrogenation product (PN) over the dimerization product (ADN). These mass transport limitations could be significantly diminished by increasing the substrate concentration to [Substrate]_high_ (Figure 4B). At this higher concentration, FE_ADN_ increased substantially and remained elevated at higher current densities compared to the [Substrate]_low_ condition. This demonstrates that improving the substrate availability at the electrode surface promotes dimer formation. Nevertheless, mass transport limitations returned as current density increased further, causing FE_PN_ to increase at the expense of FE_ADN_. A similar pattern emerged for reactions with pure CN (*x*_CN_ = 1), though with notable differences attributable to CN’s slower reactivity discussed previously. At [Substrate]_low_, CDN formation was observed, but with relatively low Faradaic efficiencies. Increasing the substrate concentration to [Substrate]_high_ enhanced CDN formation by reducing mass transport limitations, allowing for higher FE_CDN_ values across a broader range of current densities. However, as with pure AN, sufficiently high current densities eventually reintroduced mass transport limitations, causing a decrease in the FE_CDN_.

**Figure 4:**
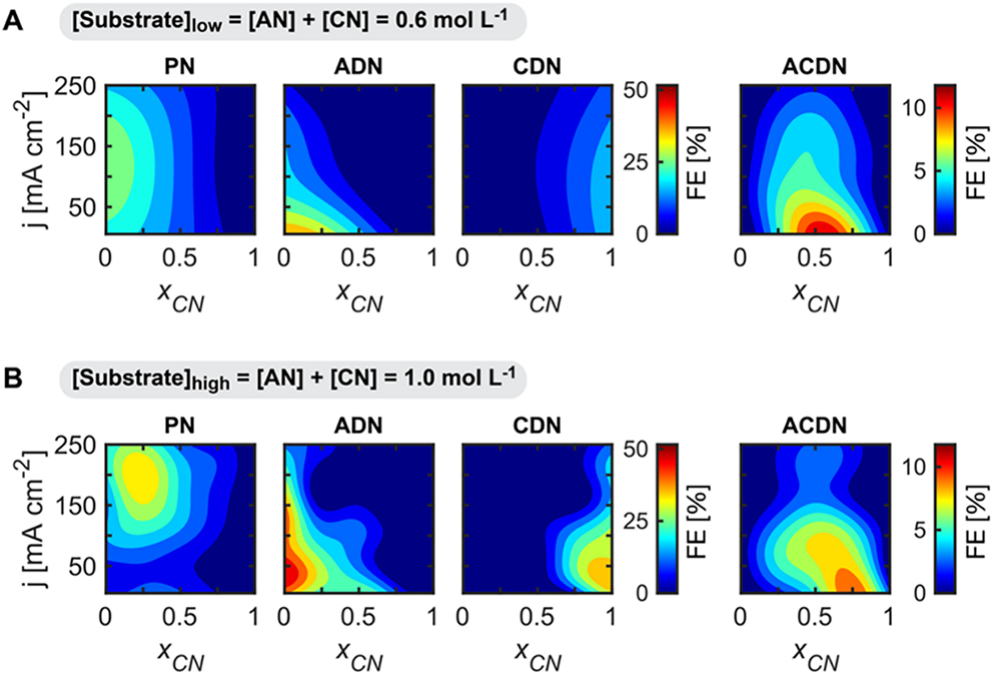
Effects of substrate concentrations and current density on product distributions. Effect of substrate composition given by CN relative molar fraction (*x*_CN_), and current density (*j*) on selectivity (Faradaic Efficiency, FE) toward AN-hydrogenation product (PN) and dimerization products (ADN, ACDN, CDN). Measurements were taken at a constant charge of 12 C. AN and CN (substrates) were kept constant at 0.6 (A) and 1.0 (B) mol L^–1^. There was negligible production of the CN hydrogenation product (BN). Electroreduction was carried out on a Cd rod in undivided parallel reactors, and electrolytes contained 0.5 mol L^–1^ Na_3_PO_4_, 0.03 mol L^–1^ EDTA, and 0.02 mol L^–1^ TBA hydroxide. The results are derived from a GPR model, trained on 120 (A) and 199 (B) experimental observations dispersed through the 2D space.

In mixed-substrate compositions, both pure AN products (PN, ADN) and pure CN products (CDN) exhibited decreasing Faradaic efficiencies as *x*_CN_ moved from their respective pure compositions (*x*_CN_ = 0 for pure AN, and *x*_CN_ = 1 for pure CN) toward equimolar conditions (*x*_CN_ = 0.5). Notably, CDN production diminished more rapidly than ADN/PN production as *x*_CN_ decreased from 1, reflecting the substantial difference in reactivity between AN and CN.

The mixed dimer ACDN displayed distinct behavior dependent on the substrate concentration, composition, and current density. Under mass transport limited conditions ([Substrate]_low_), ACDN formation reached maximum Faradaic efficiency at approximately *x*_CN_ = 0.5 across all current densities, though the magnitude decreased as current density increased. This behavior reflects the balance between reactant transport and kinetic limitations under conditions in which mass transport limitations dominate regardless of current density. When both substrates face similar transport constraints, their relative concentrations at the electrode surface mirror bulk concentrations, making *x*_CN_ = 0.5 optimal for mixed dimer formation.

When the substrate concentration increased to [Substrate]_high_, mass transport limitations were initially diminished, causing product distribution to be more strongly influenced by kinetic differences. At low current densities and equimolar conditions (*x*_CN_ = 0.5), faster AN kinetics dominated, favoring PN/ADN formation over ACDN, as the PN^•^ formation rate significantly exceeded the BN^•^ formation rate. Interestingly, the maximum FE_ACDN_ shifted to *x*_CN_ ≈ 0.75 under these conditions. At this molar ratio (∼0.25 M AN and ∼0.75 M CN), the faster kinetics of AN were offset by its slower transport due to lower bulk concentration, while the slower kinetics of CN were offset by its faster transport due to higher bulk concentration. This balance created the optimal conditions for ACDN formation. As the current density increased under [Substrate]_high_ conditions, mass transport limitations were reimposed, causing the maximum FE_ACDN_ to shift back toward *x*_CN_ = 0.5, resembling the behavior observed under [Substrate]_low_ conditions, where mass transport limitations dominated across all current densities.

Production rates followed trends consistent with our understanding of the interplay between the kinetics and mass transport (Figure S8). Maximum production rates for AN products (PN/ADN) occurred at low *x*_CN_ values and high current densities, while maximum rates for CDN were observed at high *x*_CN_ values and high current densities. Increasing substrate concentration from [Substrate]_low_ to [Substrate]_high_ alleviated mass transport limitations, enabling higher production rates for PN, ADN, and CDN at elevated current densities despite decreased selectivity. The mixed dimer (ACDN) production rate was maximized at approximately *x*_CN_ = 0.5 and higher current densities. This finding aligns with our EPR studies showing different radical formation rates between AN and CN, and it demonstrates how controlled mass transport limitations can effectively balance these inherent reactivity differences to favor desired cross-coupling products.

### Understanding Performance Drivers for Mixed-Substrate Reactions

While product distribution provides valuable information about what molecules are produced, it can be complex to analyze, particularly in mixed-substrate reactions. To better understand reactant fate and gain deeper insight into our ability to control reaction outcomes, we developed several performance metrics that address key questions: To what degree can we control the reaction? Under what conditions do we effectively utilize CN? Can we preferentially direct the reactions toward dimerization versus hydrogenation?

First, to determine if we can preferentially produce dimerization products over hydrogenation products, we introduce the Dimer Selectivity, defined as the molar fraction of dimers among all products, which is expressed as

$$\mathrm{dimer}\;\mathrm{selectivity}=\frac{2r_{\mathrm{dimerizations}}}{{2r}_{\mathrm{dimerizations}}+r_{\mathrm{hydrogenations}}}$$

where *r*_dimerizations_ and *r*_hydrogenations_ are the combined molar production rates for each set of products. As shown in Figure 5A for [Substrate]_low_, dimer production dominated at high *x*_CN_ (Dimer Selectivity is ∼1 for *x*_CN_ = 1) due to the negligible BN production (i.e. CN hydrogenation). Dimer Selectivity decreased as *x*_CN_ decreased and AN hydrogenation to PN increased, though dimers were still favored at lower current densities, where mass transport limitations were less severe. Similarly, increasing the substrate concentration to [Substrate]_high_ (Figure 5B) reduced mass transport limitations further, resulting in high Dimer Selectivity at low current densities for all *x*_CN_. This advantage diminished only as the current density increased, and mass transport limitations returned.

**Figure 5:**
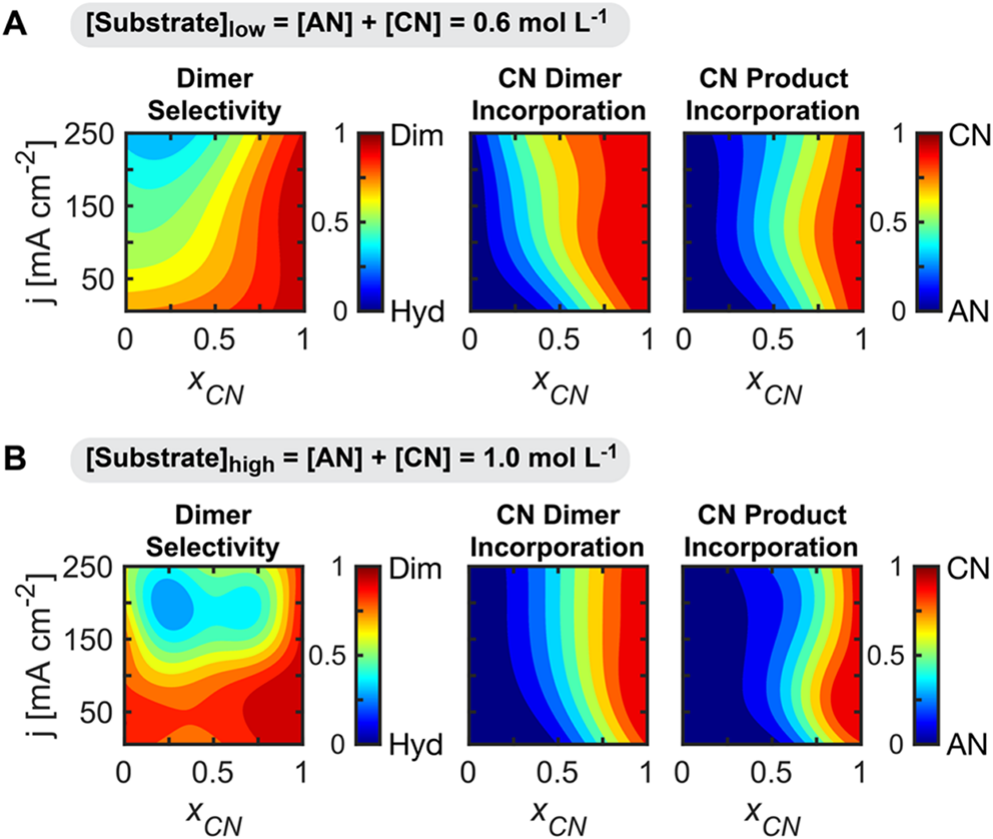
Mapping correlations between the reaction parameters and product composition metrics. Effect of substrate composition given by the CN relative molar fraction (*x*_CN_), and current density (*j*) on dimer selectivity, CN dimer incorporation, and CN product incorporation at a constant charge of 12 C. AN and CN (substrates) combined concentration was kept constant at 0.6 (A) and 1.0 (B) mol L^–1^. Electroreduction was carried out on a Cd rod in undivided parallel reactors, and electrolytes contained 0.5 mol L^–1^ Na_3_PO_4_, 0.03 mol L^–1^ EDTA, and 0.02 mol L^–1^ TBA hydroxide. The results are derived from a Gaussian process regression model, trained on 120 (A) and 199 (B) experimental observations dispersed through 2D space.

To assess which dimers are preferred under various conditions, we measured the CN Dimer Incorporation, defined as the fraction of CN monomers incorporated into the produced dimers

$$CN\;\dim er\;incorporation=\frac{r_{ACDN}+2r_{CDN}}{{2r}_{\dim erizations}}$$

where *r*_ADN_, *r*_ACDN_, and *r*_CDN_ are the production rates for each individual dimer.

Similarly, to evaluate when total products are dominated by CN versus AN, we calculated the CN Product Incorporation, which is the molar fraction of CN incorporated into all the products

$$\mathrm{CN}\;\mathrm{product}\;\mathrm{incorporation}=\frac{r_{\mathrm{ACDN}}+r_{\mathrm{BN}}+2r_{\mathrm{CDN}}}{2r_{\mathrm{dimerizations}}+r_{\mathrm{hydrogenations}}}$$

where *r*_PN_ and *r*_BN_ are the production rates for each individual hydrogenation product.

To analyze these incorporation metrics (Figure 5), it is useful to consider a hypothetical case in which both reactants exhibit similar reactivities. In such a scenario, PN^•^ and BN^•^ generation would occur at rates proportional to their bulk concentrations, and both CN Dimer Incorporation and CN Product Incorporation would match the bulk molar ratio (*x*_CN_), resulting in plots with vertical bands where color values correspond directly to *x*_CN_ values. However, as shown in Figure 5A,5B, this is not observed, and deviations from this hypothetical trend can be explained by mass transport limitations and kinetic differences.

For [Substrate]_low_ at low current densities (<75 mA cm^–2^), CN Dimer Incorporation is lower than *x*_CN_, indicating that AN dimers were favored under mild mass transport limitations. As current density increased to moderate values (100–150 mA cm^–2^), CN Dimer Incorporation approached parity with *x*_*CN*_, suggesting that mass transport limitations balanced PN^•^ and BN^•^ generation. At even higher current densities (>150 mA cm^–2^), CN Dimer Incorporation exceeded *x*_CN_, likely because hydrogenation became the dominant pathway for AN reduction under severe mass transport limitations, thereby increasing the relative BN^•^ concentration compared to PN^•^. For [Substrate]_high_, CN Dimer Incorporation favored AN dimers at low current densities, where mass transport limitations are minimal. As current density increased, reimposing mass transport limitations, CN Dimer Incorporation approached the bulk *x*_CN_, indicating that the generation of both PN^•^ and BN^•^ reached equilibrium when both substrates were mass transport limited.

CN Product Incorporation also exhibited deviations from parity with *x*_CN_, generally favoring AN products under most conditions. For [Substrate]_low_, at low current densities and *x*_CN_ < 0.5, products predominantly incorporated AN monomers. As the current density increased, AN became mass transport limited, bringing CN Product Incorporation closer to *x*_*CN*_ as both substrates are consumed at comparable rates. However, this balanced incorporation was not observed at [Substrate]_high_, as illustrated by the shift toward AN products (at *x*_CN_ = 0.5, CN Product Incorporation is <0.3 for all current densities; at *x*_CN_ = 0.75, CN Product Incorporation is between 0.5 and 0.75 for all current densities). Given the improved transport conditions for AN at this higher bulk concentration, the faster AN kinetics make it the dominant substrate across all mixed conditions.

Our comprehensive analysis reveals that product selectivity in electrochemical mixed-olefin dimerization is governed by a delicate balance between inherent kinetic differences and mass transport phenomena. While molar ratio (*x*_CN_) emerged as the primary determinant of product distribution, controlling which reactant dominates at the electrode surface, substrate concentration and current density served as powerful secondary levers that modulate mass transport limitations. Notably, we found that optimal conditions for specific products often arise from counterintuitive parameter combinations. For instance, the mixed dimer ACDN formation was maximized under conditions where faster-reacting AN was transport-limited while slower-reacting CN was not, creating a difficulty to accomplish balance between the formation of different radical intermediates. The performance metrics we developed here further demonstrate that selective production of targeted dimers remains challenging without strategic adjustment of molar ratios, particularly for mixed dimers. While these static parameter adjustments provide significant control over selectivity, we next investigated whether dynamic modulation of electrochemical conditions could further enhance our ability to precisely direct product formation in mixed-substrate systems.

### Dynamic Control of Selectivity through Pulsed Electrosynthesis

Pulsed electrosynthesis offers the potential for improved selectivity control through dynamic modulation of electron transfer rates, which can be used to balance mass transport processes. As shown in Figure 6A, we investigated time-varying pulse sequences–alternating between cathodic current density (*j*_c_) for duration (*t*_c_) and zero-current rest periods (*t*_r_)–to strategically manipulate substrate concentration at the electrode interface. During active pulses, substrate reduction generates radicals at rates proportional to both *j*_c_ and local concentration, while the subsequent rest periods halt Faradaic reactions, allowing AN and CN near-electrode concentrations to regenerate. Our group previously demonstrated that this pulsed approach effectively overcomes mass transport limitations in ADN electrosynthesis,^40^ achieving significantly increased production rates at high current densities by precisely controlling reactant availability in the electrical double layer (EDL).

**Figure 6:**
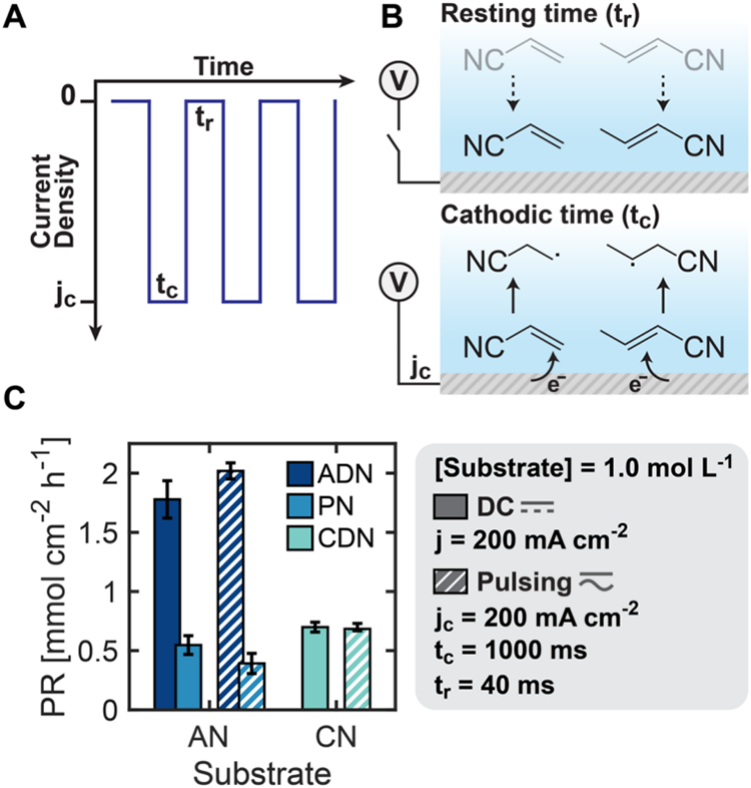
Tuning production rates through cathodic pulse sequencing. (A, B) Pulse chronopotentiometry waveform, highlighting the resting current density (0), cathodic current density (*j*_c_), resting time (*t*_r_), and cathodic time (*t*_c_). Schematic representation of the effects of cathodic and resting times on the near-electrode environment. (C) Production rate of ADN and PN when using the AN substrate and CDN when using the CN substrate for direct current (DC) and pulse chronopotentiometry electrolysis. There was negligible production of the CN hydrogenation product (BN). Electroreduction was carried out on a Cd rod in undivided parallel reactors, at 200 mA cm^–2^ current density for DC and 200 mA cm^–2^ *j*_c_, 1000 ms *t*_c_, and 40 ms *t*_r_ for pulsing measurements. Electrolytes contained 1.0 mol L^–1^ substrate (AN or CN), 0.5 mol L^–1^ Na_3_PO_4_, 0.03 mol L^–1^ EDTA, and 0.02 mol L^–1^ TBA hydroxide.

Figure 6B illustrates the effect of pulsed electrosynthesis on product distributions using either the AN or CN-only substrate. With pulse conditions of *j*_c_ = 200 mA cm^–2^, *t*_c_ = 1000 ms, and *t*_r_ = 40 ms, ADN production increased by 14% compared with direct current operation at the same current density, while the undesired PN production decreased by 28%. This improvement stems from the periodically renewed AN concentration at the electrode surface during rest periods (*t*_r_), effectively mitigating further protonation to PN and favoring radical coupling to form ADN. In contrast, when using CN as a substrate, we observed minimal difference in hydrodimer (CDN) production between DC and pulsed operation. This contrasting behavior likely occurs because CN shows negligible hydrogenation to BN. Because CN’s electrochemical pathway is not compromised by competing hydrogenation to BN at low near-electrode concentrations, the pulsed enhancement of local CN concentration provides little benefit. The slight decrease in CDN production rate under pulsed conditions reflects only the shorter effective reaction time, an effect minimized in our experiments using *t*_c_ values 25 times greater than *t*_r_. Experiments with multiple pulse sequences revealed how the three pulsing parameters (*j*_c_, *t*_c_, and *t*_r_) distinctly influence production rates in the electrolysis of AN or CN-only substrates, with hydrodimers showing optimal behavior at specific combinations of pulse duration and magnitude (Figures S9–S10). Next, we leveraged these insights to address the more complex challenge of controlling selectivity in mixed AN/CN systems through strategic application of pulsed electrolysis.

We investigated how pulsed operation could strategically manipulate the mass transport and reaction kinetics in AN/CN mixtures to control the dimer distribution. By systematically varying *j*_c_, *t*_c_, and *t*_r_ while maintaining a total substrate concentration of 1.0 mol L^–1^ and *x*_CN_ of 0.6, we mapped the effects of pulse parameters on product distributions. Figure 7 presents two-dimensional slices (varying *t*_c_ and *j*_c_) at fixed *t*_r_ values of 1 and 100 ms. The complete three-dimensional CN Dimer Incorporation and Dimer Selectivities across all *t*_r_ values can be found in (Figures S11–S12). At short *t*_r_ (1 ms), ADN achieves the highest maximum production rate among the three dimers—a direct consequence of maximized Faradaic currents under these conditions. This regime makes dimer formation primarily dependent on radical generation rates, where radicals produced from AN dominate those produced from CN according to our EPR spectroscopy studies. This selectivity behavior becomes particularly evident at very short *t*_c_, where insufficient AN depletion leads to AN accumulation at the electrode interface, strongly promoting ADN formation while suppressing CN-containing dimers. The selectivity progressively shifts toward ACDN and CDN at longer *t*_c_ values, as extended reduction periods modulate the near-electrode concentration ratio by preferentially depleting AN, ultimately making CDN the predominant dimer at *t*_c_ = 1000 ms. When extending *t*_r_ to 100 ms, we observed shift toward longer values of *t*_c_ to maximize production for all dimers, as maintaining an optimal active-to-resting ratio requires longer active periods to compensate for the extended resting phase. Notably, at *t*_r_ = 100 ms, all three dimers reach comparable maximum production rates (∼0.2 mmol cm^–2^ h^–1^), indicating that the shorter *t*_r_ of 1 ms is insufficient for significant CN concentration regeneration at the electrode surface. These results demonstrate how pulse parameters can be precisely tuned to expose the reaction to different transport limitation regimes, thereby providing control over competitive radical coupling pathways and their resulting product distributions.

**Figure 7:**
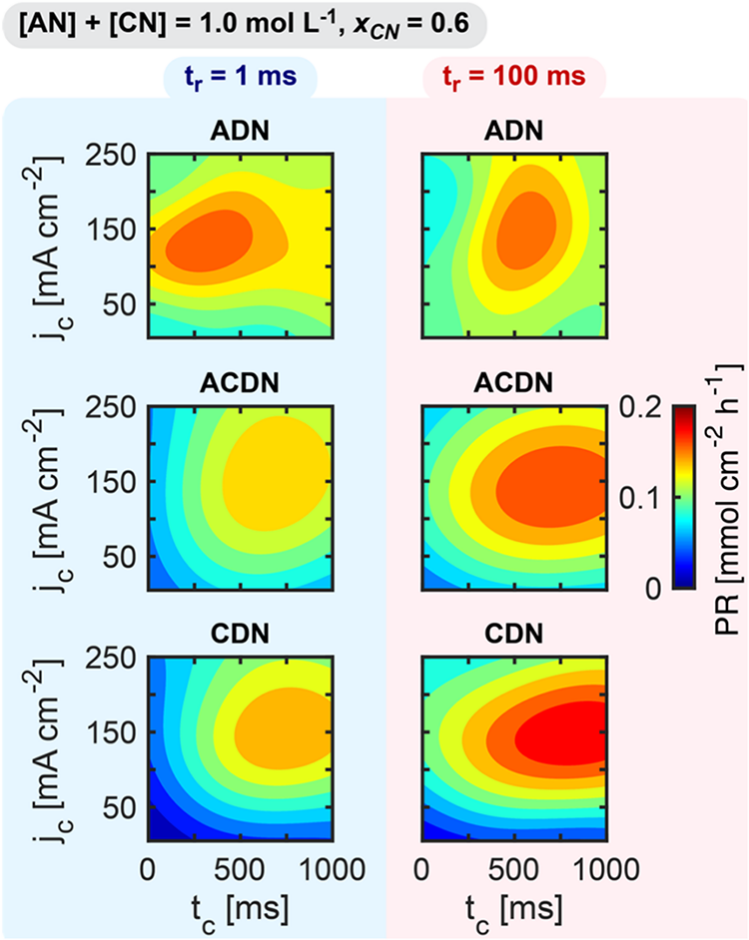
Pulse sequence effects on mixed-substrate dimer production rates. 2D slices of the 3D surrogate model of the effect of *j*_c_, *t*_r_, and *t*_c_ on the production rate of dimers (ADN, ACDN, and CDN) keeping constant *t*_r_ of 1 and 100 ms. Pulsed electroreduction was carried out on a Cd rod in undivided parallel reactors, at a constant cumulative cathodic charge transferred of 12 C. Electrolytes contained 0.4 mol L^–1^ AN, 0.6 mol L^–1^ CN, 0.5 mol L^–1^ Na_3_PO_4_, 0.03 mol L^–1^ EDTA, and 0.02 mol L^–1^ TBA hydroxide. The results are derived from a Gaussian process regression model, trained on 50 experimental observations dispersed through the 3D space.

Shifting our analysis from individual dimer production rates to compositional metrics, we evaluated the incorporation of CN dimers (the fraction of CN monomers in the product mixture) across varying *x*_CN_ values at a constant bulk concentration (1.0 mol L^–1^). Figure 8 illustrates how CN dimer incorporation varies as a function of current density (*j*_c_) at fixed pulse durations (*t*_c_ and *t*_r_), with separate curves representing different *x*_CN_ values. The complete 3D parameter-response maps from which these data were derived are presented in Figure S11. As expected, higher *x*_CN_ values consistently resulted in greater CN Dimer Incorporation. A clear trend emerged across all substrate compositions: CN Dimer Incorporation increased at higher *j*_c_, likely due to an intensified rate of consumption of AN, and approached (but did not surpass) the bulk *x*_CN_. At low current densities, pulsing effects were minimal since AN was not mass transport limited, resulting in similar trends to DC operation, where CN Dimer Incorporation was lower than *x*_CN_ because the faster PN^•^ formation favored ADN dimer production.

**Figure 8:**
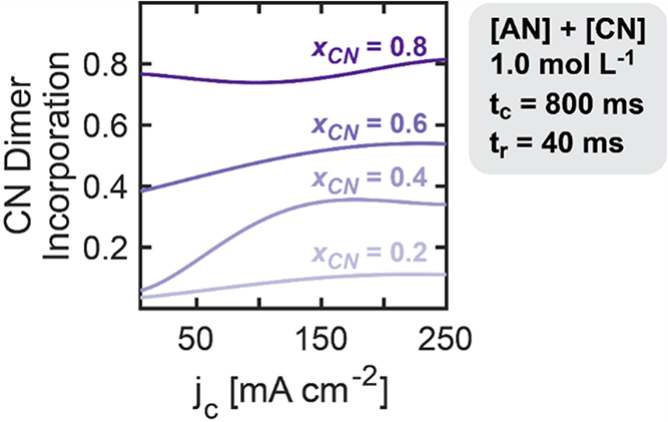
Active current effects on CN dimer incorporation across substrate compositions. Effect of *j*_c_ on the incorporation of CN in dimers keeping constant *t*_c_ of 800 ms and *t*_r_ of 40 ms at varied CN relative molar fraction (*x*_CN_). Pulsed electroreduction was carried out on a Cd rod in undivided parallel reactors at a constant cumulative cathodic charge transferred of 12 C. Electrolytes contained 1.0 mol L^–1^ substrate (AN and CN combined), 0.5 mol L^–1^ Na_3_PO_4_, 0.03 mol L^–1^ EDTA, and 0.02 mol L^–1^ TBA hydroxide. The results are derived from a Gaussian process regression model, trained on 54 (*x*_CN_ = 0.2), 52 (*x*_CN_ = 0.4), 50 (*x*_CN_ = 0.6), and 55 (*x*_CN_ = 0.8) experimental observations dispersed through 3D space.

## Conclusions

In this study, we established a mechanistic framework for understanding and controlling the selectivity in mixed-substrate electroreduction by systematically investigating the interplay between inherent kinetic differences and mass transport limitations. Through the implementation of an HTE workflow, we successfully conducted hundreds of reactions testing combinations of reaction conditions to establish relationships among the composition of AN/CN mixtures, current densities, and product distributions.

Our findings reveal that product selectivity in mixed-substrate electroreduction is governed by a delicate balance between substrate-specific reaction kinetics and mass transport phenomena. The structural difference between AN and CN, a simple methyl substitution, creates profound reactivity differences that manifest in both radical formation rates and coupling pathways. EPR spectroscopy confirmed that radical formation from AN occurs more rapidly than that from CN, explaining the preferred formation of ADN under reaction-limited conditions. Notably, CN showed negligible hydrogenation to BN across all experimental conditions, highlighting how subtle molecular modifications can alter the competing reaction pathways.

We demonstrated that product distributions can be precisely controlled through the strategic manipulation of three key parameters: substrate concentration, molar fraction, and current density. While the molar fraction emerged as the primary determinant of product distribution, substrate concentration and current density served as powerful secondary levers that modulate the balance between reaction and mass transport limitations. This allowed us to create specific microenvironments at the electrode surface where counterintuitive reactivity patterns emerged. Most notably, the mixed dimer ACDN formation was maximized when faster-reacting AN was transport-limited, while slower-reacting CN was transport-abundant, creating optimal conditions for cross-coupling.

Our implementation of pulsed electrosynthesis provided unprecedented dynamic control over the electrode microenvironment, enabling the selective modulation of product distributions beyond what is possible with static parameter adjustment. By strategically varying pulse parameters (*j*_c_, *t*_c_, and *t*_r_), we created transient concentration profiles that temporarily equilibrated the availability of otherwise kinetically distinct substrates. This approach proved particularly beneficial for controlling the formation of mixed dimers, which require balanced radical concentrations. The complex responses to pulse parameters underscore how electrochemical reactions can be controlled not just by steady-state kinetic considerations but also through the deliberate manipulation of transport phenomena.

These findings highlight the importance of controlling the concentrations of reactive organic species for selective electrosynthesis, particularly in the presence of mixtures. The mechanistic understanding of how kinetics and mass transport interact in mixed-substrate systems provides a framework that extends beyond the AN/CN model system. The principles demonstrated here, strategic modulation of mass transport limitations and precise control of near-electrode environments, could be directly applied to more complex electro-organic processes such as biomass conversion, where multiple reactive species compete at the electrode surface. Furthermore, our approach of balancing kinetics and mass transport through parameter tuning offers a pathway toward adaptive chemical manufacturing processes capable of handling impure feedstocks, potentially reducing separation costs and enabling the valorization of inexpensive waste streams.

## Experimental Methods

### Chemicals and Materials

Acrylonitrile (AN, >99%), propionitrile (PN, 99%), adiponitrile (ADN, 99%), crotononitrile (CN, mixture of cis and trans 99%), sodium phosphate (Na_3_PO_4_, 96%), ethylenediaminetetraacetic acid disodium salt dihydrate (EDTA·2H_2_O, ≥98.5%), deuterium oxide (D_2_O, 99.8% atom % D), toluene-*d*_8_ (99.6% atom % D), dichloromethane (>99.8%), and sulfuric acid (H_2_SO_4_, 95–97%) were purchased from Sigma-Aldrich. Toluene was purchased from VWR. Butyronitrile (BN, 99%) and Cadmium foil (0.5 mm thick, 99.9975%) were purchased from Fisher Scientific. Tetrabutylammonium hydroxide (TBA, 40% wt % in H_2_O) and ethylene carbonate (EC, >99.0%) were purchased from TCI Chemicals. Hexane-1,3,6-tricarbonitrile (trimer, 98%) was purchased from Ambeed (IL, USA). Platinum gauze (52 mesh woven from 0.1 mm dia wire, 99.9%) was purchased from Alfa Aesar. Platinum and cadmium rods (1.6 mm diameter and 31.2 mm length) were purchased from Analytical Sales and Services (NJ, USA). Nafion 117 was purchased from Ion Power, Inc. Hydrogen calibration gases (310 and 1000 ppm, balance air) were purchased from GASCO (IL, USA) and DOD Technologies (IL, USA), respectively. Argon gas was purchased from Airgas (PA, USA). Deionized (DI) water was used for all experiments in this work.

### HTE Electrochemical Characterization

High-throughput experimentation (HTE) was carried out in a 24-well reactor for parallel screening of electrochemically relevant parameters (HTe̅Chem) purchased from Analytical Sales and Services (NJ, USA). Electrolyte preparation for HTE was achieved using an Opentrons OT-2 pipetting robot (NY, USA). Reactions were performed using constant current, calibrated DC power supply, four-output multirange, 420W combined output, and vigorous stirring (3000 rpm). Chronopotentiometry (CP) experiments were carried out by setting the desired current, and the time duration was set to maintain the total charge transferred constant (200 mA min) along the different current densities evaluated. Low conversion of the substrate was chosen to maintain a relatively constant bulk concentration of the species throughout the reaction. A constant electrolyte volume of 0.5 mL was used, and the electrodes surface area was 0.55 cm^2^. The electrodes consisted of a cadmium rod as the cathode and a platinum rod as the anode. All electrochemical characterizations in this study used an electrolyte containing 0.5 mol L^–1^ Na_3_PO_4_ for pH control and improved conductivity, 0.03 mol L^–1^ EDTA to prevent undesired metal cation electrodeposition, and 0.02 mol L^–1^ TBA hydroxide for selectivity enhancement and cathode corrosion prevention.

### H-Cell Electrochemical Characterization

A 3-electrode setup was used to collect and quantify gas products on cathodic half-cell reactions. Electrochemical Impedance Spectroscopy (EIS) and chromatopotentiometry (CP) experiments were performed using a BioLogic VSP-300 potentiostat equipped with a ±1 A/±48 V booster. A machined Teflon H-cell with the cathode side sealed to the atmosphere and equipped with an argon purge was used to aid in the collection of gas products (Figure S4). Argon flowed at a rate of 26 sccm into the reactor. A cold trap was incorporated downstream of argon to condense volatile products. Gas products were collected using a Tedlar sample bag. The anolyte and catholyte were separated with a Nafion N117 membrane and sealed between 2 Viton gaskets, avoiding the deposition of metal ions from the anode on the cathode surface and oxidation of organic molecules. The electrodes consisted of cadmium foil of 3 cm^2^ as the cathode, a platinum mesh as the anode, and an Ag/AgCl electrode in saturated KCl solution as the reference electrode. The cadmium electrode was stored in an aqueous electrolyte when not in use. Electrodes not stored in this way could affect the reactor performance, possibly due to the formation of undesired surface oxides. EIS was used to characterize the resistance of the cell and correct the electrode potential for *iR* losses. Before each experiment, a fresh stock solution of 0.5 M Na_3_PO_4_, 0.03 M EDTA, and 0.02 M TBA–OH in H_2_O was mixed with AN and/or CN with the desired substrate composition. The anolyte consisted of a 1 mol L^–1^ solution of sulfuric acid. Constant catholyte and anolyte volumes of 10 mL were vigorously stirred with 4 mm × 8 mm microstir bars.

### Chemical Quantification

Liquid product mixtures were analyzed by using the following procedure. After each experiment, the aqueous electrolyte mixture was added to a separatory funnel and mixed with toluene for liquid–liquid extraction of the organic molecules. The organic phase was then analyzed using a Shimadzu gas chromatographer equipped with a mass spectrometer (GCMS-QP2010) and calibration curves for all molecules of interest are shown in Figure S13. For HTE, the extraction procedure was automated using an Opentrons OT-2 pipetting robot. Standard solutions of 3,4-dimethyladiponitrile (CDN) and 3-methyladiponitrile (ACDN) were prepared by electrolysis of AN/CN mixtures, followed by purification via liquid–liquid extraction with dichloromethane and rotary evaporation. The purified products were dissolved in toluene and quantified via ^1^H NMR using a Bruker Avance III 400 NMR Spectrometer, then used to construct GC calibration curves. Spectra were obtained with a delay time of 8 s, 16 scans per measurement, and without solvent suppression. The samples consisted of a 50% organic mixture in toluene and 50% toluene-*d*_8_, with the addition of a solution of ethylene carbonate for product quantification. See Figure S14 for sample NMR spectra. Hydrogen gas quantification was performed using an Agilent 990 Micro GC instrument equipped with a MolSieve 5A column. Acquisition parameters were as follows: injection temperature 110 °C, injection time 40 ms, column temperature 80 °C, argon carrier gas, and run time 80 s. A standard gas mixture containing H_2_ was used as a reference.

### Electron Paramagnetic Resonance Measurements

The following electrochemical procedure was used to capture and detect the free radicals generated during the electroreduction of AN and/or CN carried out in an aqueous electrolyte. Electrolysis was carried out at −200 mA cm^–2^ with 424 mg of DMPO to achieve detectable concentrations of radical species for EPR measurements. After the electrolysis, an electrolyte sample of constant volume of 25 μL was extracted with a quartz capillary for immediate EPR tests. The EPR experiments were performed through a Bruker ELEXSYS E500 EPR. Eight accumulated scans were acquired to measure DMPO adducts.

## Supplementary Material



## Notes

Building GPR surrogate models, H-cell design for detailed reaction characterization, direct current and pulsed electrolysis supporting data, and liquid chemical quantification via GC-MS and ^1^H NMR (PDF)

## Abbreviations

ACDN: 3-methyladiponitrile
ADN: adiponitrile
AN: acrylonitrile
BN: butyronitrile
CDN: 3,4-dimethyladiponitrile
CN: crotononitrile
CP: chronopotentiometry
DC: direct current
DI: deionized
DMPO: 5,5-dimethyl-1-pyrroline N-oxide
EC: ethylene carbonate
EDL: electrical double layer
EDTA: ethylenediaminetetraacetic acid disodium salt dihydrate
EIS: electrochemical impedance spectroscopy
EPR: electron paramagnetic resonance
FE: faradaic efficiency
GC: gas chromatography
GCMS: gas chromatography–mass spectrometry
GPR: gaussian process regression
HTE: high-throughput experimentation
NMR: nuclear magnetic resonance
PN: propionitrile
TBA: tetrabutylammonium
