# Supplementary material for: Controlling Selectivity in Electrochemical Conversion of Organic Mixtures through Dynamic Control of Electrode Microenvironments
Source: J Am Chem Soc. 2025 Oct 4;147(41):37576–86. doi: 10.1021/jacs.5c12445 (PMC12532195; doi:10.1021/jacs.5c12445)
Supplement: Supplementary file 1 [file ja5c12445_si_001.pdf]

Supporting Information

Controlling Selectivity in Electrochemical Conversion of Organic Mixtures through Dynamic Control of Electrode Microenvironments

Ricardo Mathison,[a] Elina Rani,[a] Amelia M. Rose,[a] Fjona Prendi,[a] Casey K. Bloomquist,[a] Miguel A. Modestino[a]
[a] Department of Chemical and Biomolecular Engineering, New York University, Brooklyn, NY 11201, USA

Corresponding author: Miguel A. Modestino
Email: modestino@nyu.edu

This PDF file includes:

Figures S1 to S15
Tables S1 to S2

Building GPR surrogate models

Gaussian process regression (GPR) surrogate models were built to establish relationships between reaction parameters and performance, as determined by product mixture composition. Our systematic workflow, which was used to build all 1D, 2D, and 3D models in this study, is illustrated in Figure S1 using the product distribution metric at 1.0 M substrate concentration as an example. Below, we detail the step-by-step process following this workflow.

Parameter Grid Design. Boundaries were defined for each variable. Points within these boundaries were combinatorially selected using Hammersley sampling,[1] a pseudo-random technique, along with a set of boundary condition points to ensure comprehensive exploration of the parameter space. Figure S2 shows the points at all experimental conditions tested in this study.

Experimental Data Collection. Experiments were conducted using the HTE setup under all selected reaction conditions, with liquid products quantified to calculate reaction performance metrics. Results were plotted with a defined 'colorbar' to establish relationships between the data and displayed colors.

Surrogate Model Development. Data was input to a GPR in MATLAB using the 'fitrgp' function, with reaction conditions as input features and reaction performance metrics as output values. The function utilized GPR to learn relationships in the data. The resulting model enabled data visualization through prediction of values for new inputs, effectively capturing underlying trends and uncertainties in the data.

Trend Analysis. The GPR model's prediction of relationships between reaction parameters and product distributions was examined and analyzed using heat-map visualizations.

Error Assessment. The uncertainty in predictions was assessed using two methods. First, predicted and experimentally measured performance metrics were compared to calculate their root mean squared error (RMSE). Second, the standard deviation was obtained from the GPR model, with larger values indicating areas requiring more data or investigation. For regions showing high uncertainty, additional experiments were conducted to evaluate impact on overall model performance. The HTE setup's reproducibility was evaluated by conducting identical conditions across 16 separate reactors, with experimental uncertainty shown in Figure S3. Tables S1 and S2 present the median standard deviation and RMSE between predicted and measured metrics for all models.

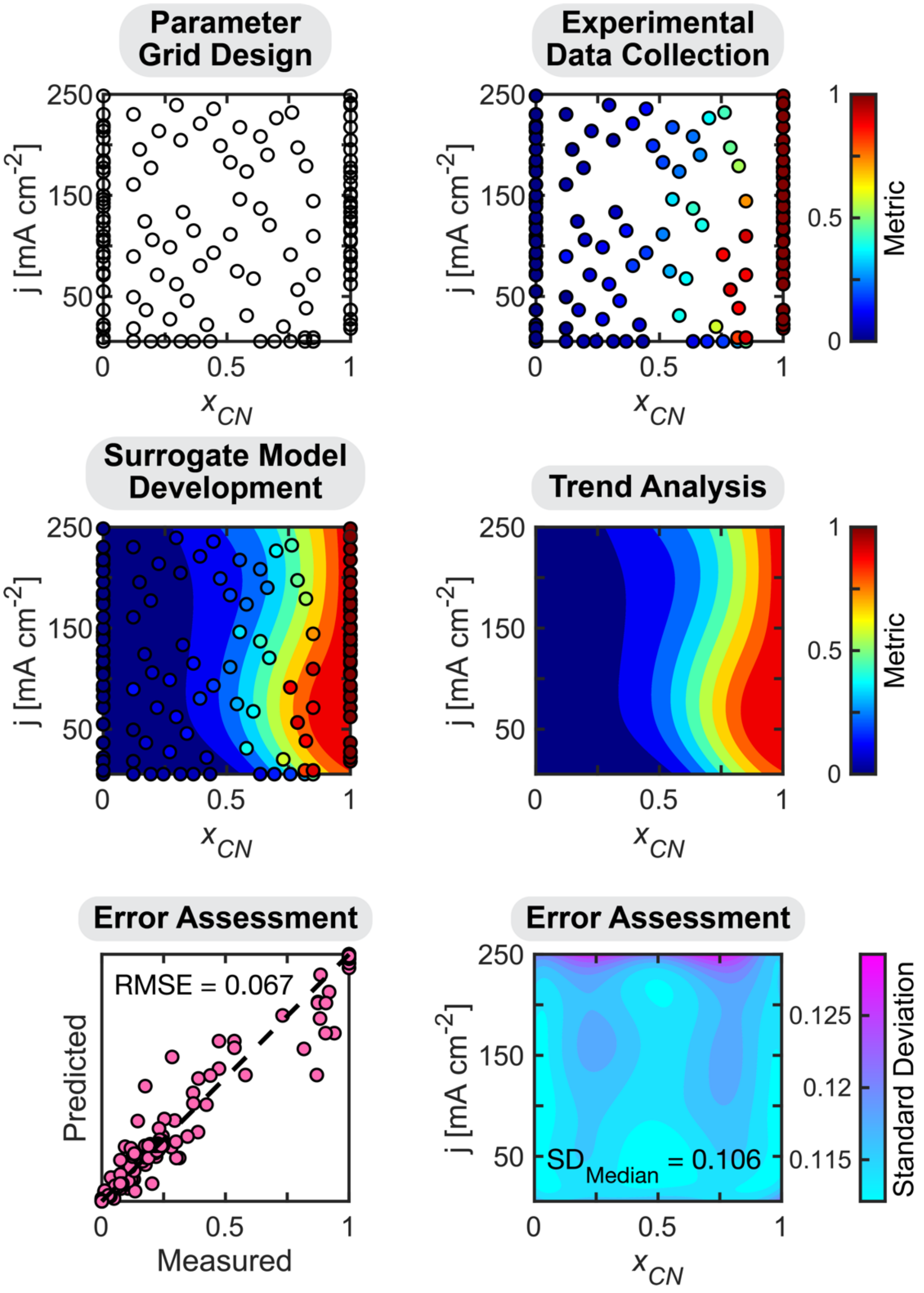

Figure S1. Process for building data-driven surrogate models.

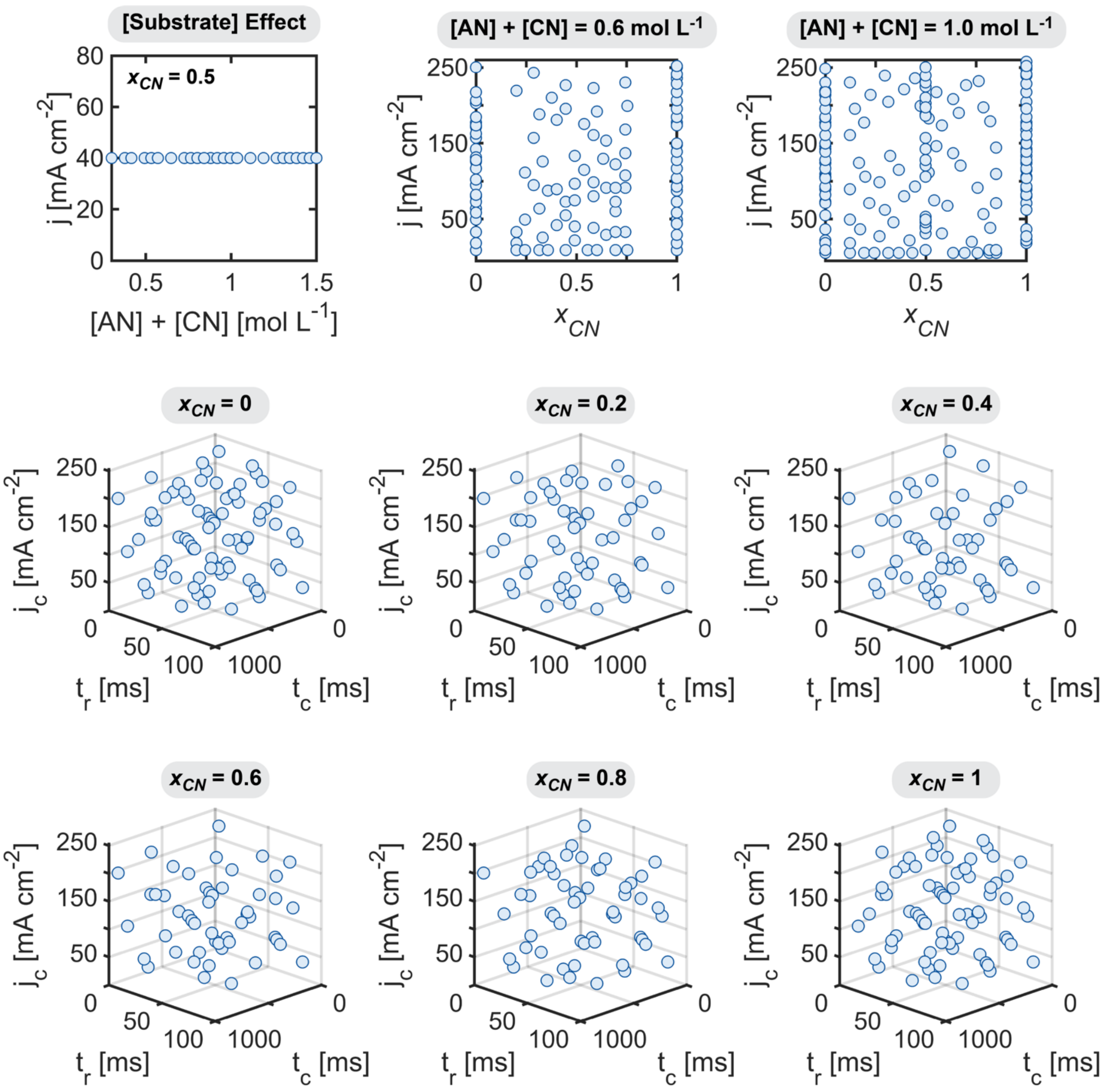

Figure S2. Pseudo-random experimental conditions selected using Hammersley sampling. Experimental data is collected by performing electrochemical reactions at the selected conditions and fed to GPR surrogate models to produce the maps shown in this study for: Substrate concentration effects (top left), current density and substrate composition effects under direct current electrolysis (top center-right), and pulsed electrolysis experiments (bottom).

Table S1. Error assessment on data-driven surrogate models for DC experiments. Error is represented by the median standard deviation (SD_{Median}) of the surrogate models and by the root mean squared error (RMSE) of the predictions compared to the experimental data.

Section	Experimental Conditions	Metric	SD_{Median}	RMSE
Substrate concentration effect	40 mA cm^{-2} x_{CN} = 0.5	ADN FE	3.816	5.203
		PN FE	3.690	1.916
		CDN FE	1.043	0.703
		ACDN FE	0.664	1.914
j and x_{CN} effects (Direct current)	$[Substrate]_{low}$ [AN] + [CN] 0.6 mol L^{-1}	PN FE	3.398	6.589
		ADN FE	3.358	6.841
		CDN FE	2.051	3.079
		ACDN FE	2.141	1.533
		PN PR	0.093	0.146
		ADN PR	0.113	0.151
		CDN PR	0.046	0.075
		ACDN PR	0.023	0.020
		Dimer Selectivity	0.083	0.102
		CN Dimer Incorporation	0.063	0.064
		CN Product Incorporation	0.043	0.056
	$[Substrate]_{high}$ [AN] + [CN] 1.0 mol L^{-1}	PN FE	4.542	4.473
		ADN FE	5.007	6.578
		CDN FE	2.952	5.714
		ACDN FE	2.565	1.668
		PN PR	0.111	0.113
		ADN PR	0.140	0.160
		CDN PR	0.046	0.123
		ACDN PR	0.057	0.031
		Dimer Selectivity	0.160	0.088
		CN Dimer Incorporation	0.114	0.082
		CN Product Incorporation	0.116	0.064

Table S2. Error assessment on data-driven surrogate models for pulsing experiments. Error is represented by the median standard deviation (SD_{Median}) of the surrogate models and by the root mean squared error (RMSE) of the predictions compared to the experimental data.

Section	Experimental Conditions	Metric	SD_{Median}	RMSE
Pulsed electrolysis	$x_{CN} = 0$	ADN PR	0.193	0.231
	$x_{CN} = 0$	PN PR	0.091	0.115
	$x_{CN} = 1$	CDN PR	0.190	0.194
	$x_{CN} = 0.6$	ADN PR	0.046	0.034
	$x_{CN} = 0.6$	ACDN PR	0.036	0.028
	$x_{CN} = 0.6$	CDN PR	0.038	0.027
	$x_{CN} = 0.2$	CN Dimer Incorporation	0.026	0.025
	$x_{CN} = 0.4$		0.065	0.049
	$x_{CN} = 0.6$		0.070	0.067
	$x_{CN} = 0.8$		0.065	0.051
	$x_{CN} = 0.2$	Dimer Selectivity	0.064	0.058
	$x_{CN} = 0.4$		0.037	0.033
	$x_{CN} = 0.6$		0.051	0.048
	$x_{CN} = 0.8$		0.058	0.051

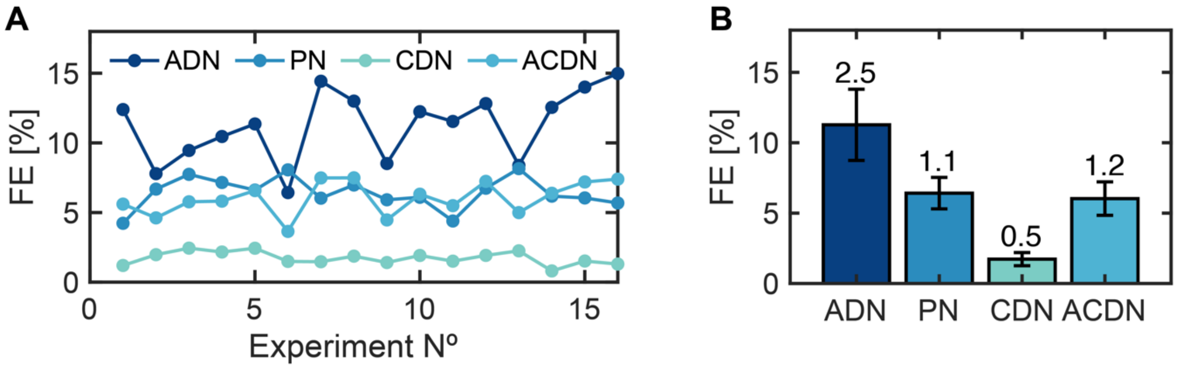

Figure S3. Reproducibility test of high-throuput parallel electrochemical reactor. (**A**) Selectivities towards organic products on 16 separate reactors performed on the same experimental run under the same experimental conditions: Substrate concentration of 1.0 mol L^{-1}, CN relative molar fraction (x_{CN}) of 0.5, and current density of 50 mA cm^{-2}. Electroreduction was carried on a Cd rod in undivided parallel reactors, and electrolytes contained 0.5 M Na_3PO_4, 0.03 M EDTA, and 0.02 M TBA hydroxide. (**B**) Mean and standard deviation of selectivities towards organic products, showing the experimental uncertainty of the parallel electrosynthesis setup.

H-Cell design for detailed reaction characterization

Despite the fact that the high-throughput experiments showed lower selectivities and production rates towards ADN compared to the 80%+ selectivities documented in other electrochemical cell configurations,[2-5] we validated our findings through comparative H-cell experiments. The performance difference is attributed to limited convection in our HTE reactors affecting mass transport conditions, yet the observed trends translate well to the H-cell experiments (Figure S7). Specifically, dimer selectivity increases with x_{CN} due to reduced BN production, while both CN dimer and product incorporation depend strongly on *[Substrate]*, with higher concentrations favoring AN-containing products. These insights provide valuable understanding of electroreduction behavior in mixed substrates regardless of the experimental setup used.

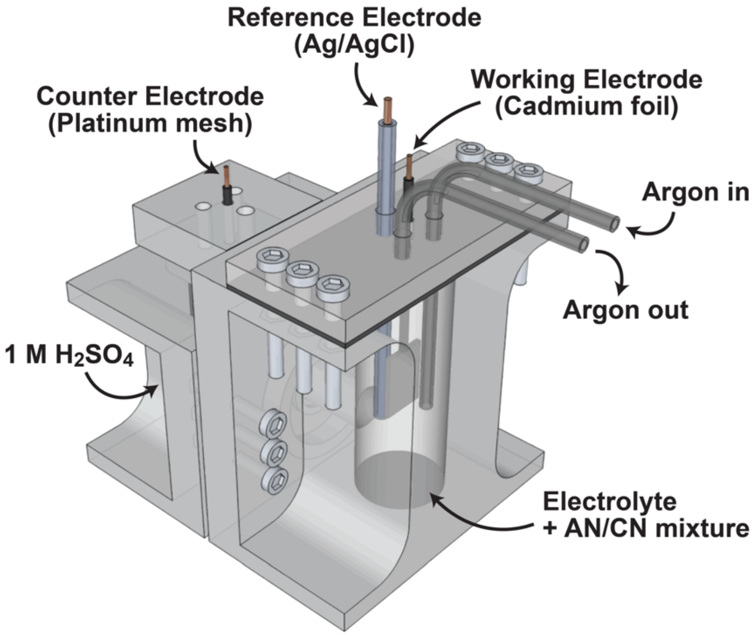

Figure S4. H-cell reactor design for detailed characterization. This design allowed for liquid and gas product collection.

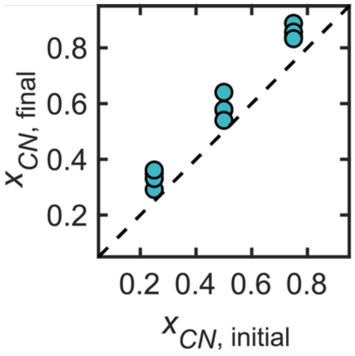

Figure S5. Substrate conversion for H-cell electrolysis. Substrate composition given by CN relative molar fraction (x_{CN}) before and after electroreduction of mixtures of AN and CN. Substrate concentration was 0.4 and 1.0 mol L^{-1}, and current density was 40 and 150 mA cm^{-2}. Electroreduction was carried on a Cd foil in a divided reactor, and electrolytes contained 0.5 M Na_3PO_4, 0.03 M EDTA, and 0.02 M TBA hydroxide.

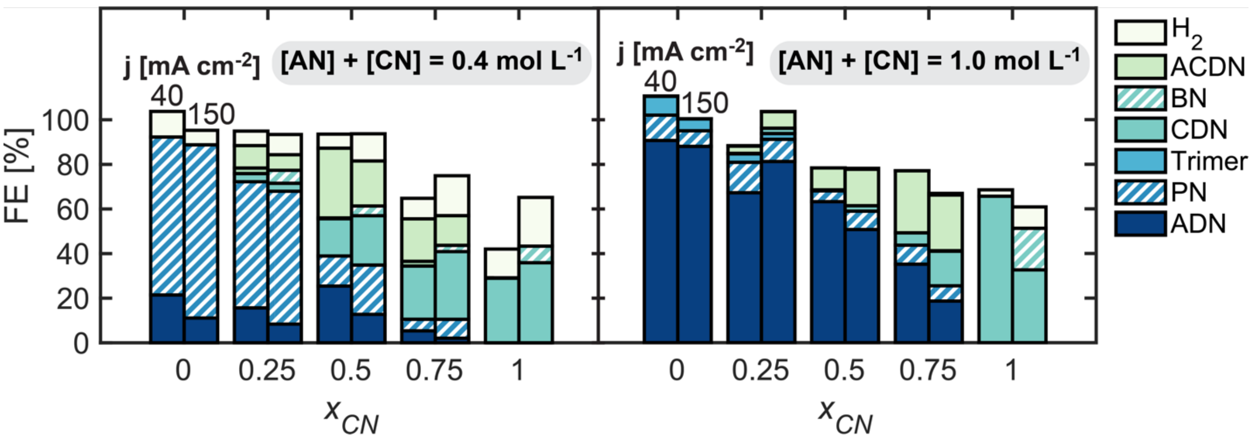

Figure S6. Product distributions for H-cell electrolysis. Selectivities towards hydrogen gas and dimerization and hydrogenation products for AN and CN electroreduction given by Faradaic efficiency (FE) as a function of CN relative molar fraction (x_{CN}), substrate concentration (*[AN] + [CN]*), and current density (*j*). Electroreduction was carried on a Cd foil in a divided reactor, and electrolytes contained 0.5 mol L^{-1} Na_3PO_4, 0.03 mol L^{-1} EDTA, and 0.02 mol L^{-1} TBA hydroxide.

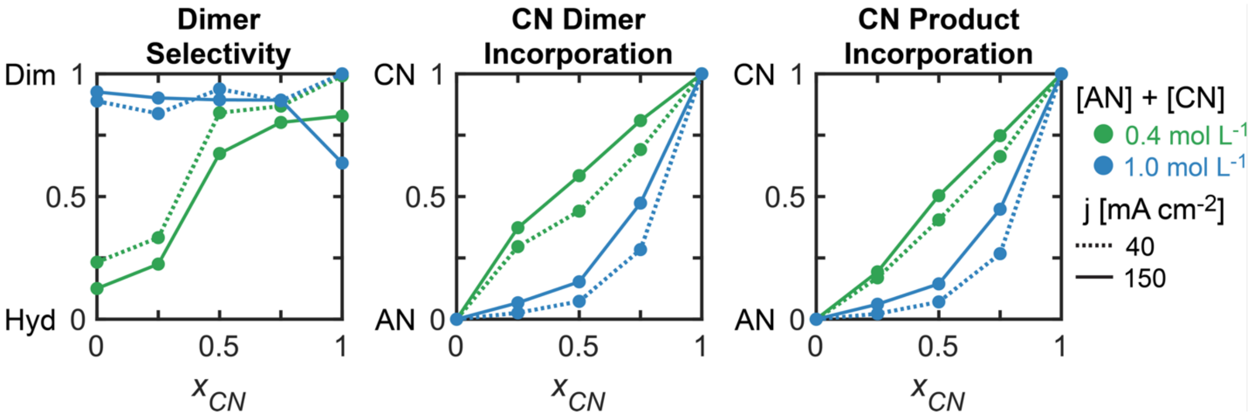

Figure S7. Product composition metrics for H-cell electrolysis. Dimer selectivity, dimer distribution, and product distribution as a function of CN relative molar fraction (x_{CN}), substrate concentration (*[AN] + [CN]*), and current density (*j*). Electroreduction was carried on a Cd foil in a divided reactor, and electrolytes contained 0.5 M Na_3PO_4, 0.03 M EDTA, and 0.02 M TBA hydroxide.

Direct Current Electrolysis Supporting Data

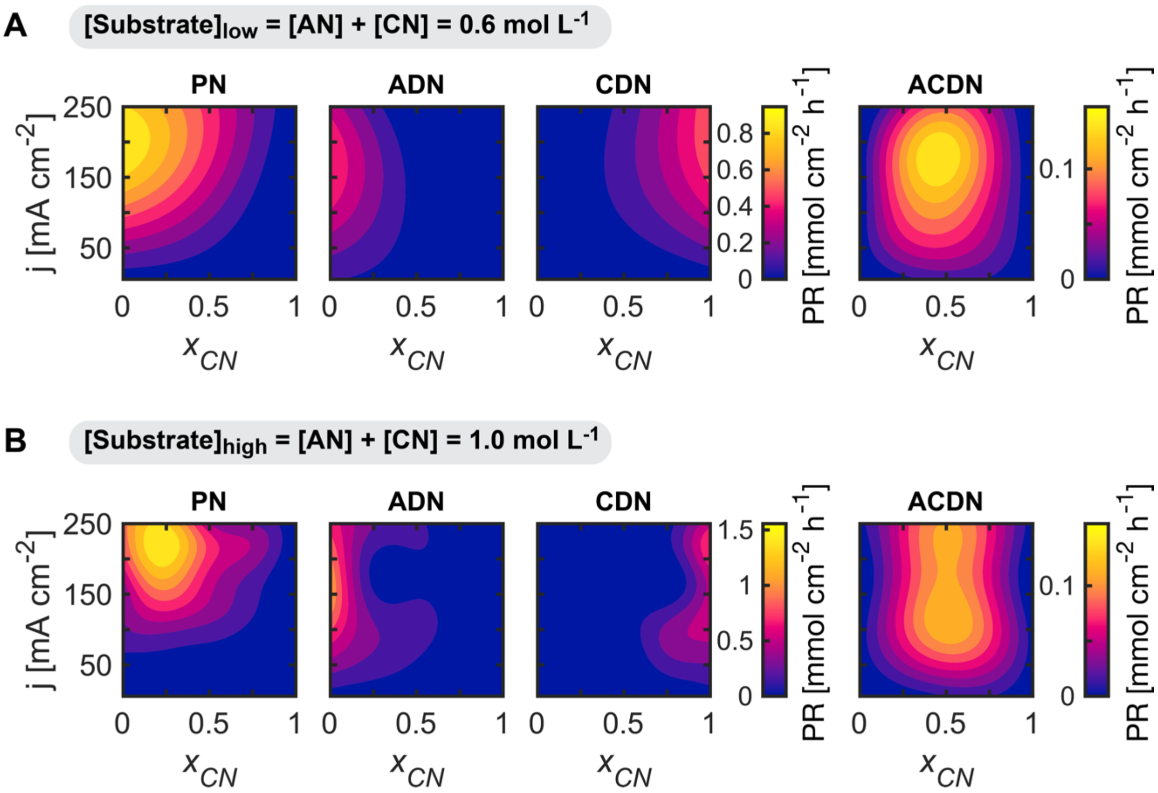

Figure S8. Effects of substrate concentrations and current density on production rates. Effect of substrate composition given by CN relative molar fraction (x_{CN}), and current density (j) on production rates (PR) toward AN-hydrogenation product (PN) and dimerization products (ADN, ACDN, CDN). Measurements were taken at a constant charge of 12 C. AN and CN (substrates) combined concentration was kept constant at 0.6 (**A**) and 1.0 (**B**) mol L^{-1}. There was negligible production of CN hydrogenation product (BN). Electroreduction was carried on a Cd rod in undivided parallel reactors, and electrolytes contained 0.5 mol L^{-1} Na_3PO_4, 0.03 mol L^{-1} EDTA, and 0.02 mol L^{-1} TBA hydroxide. The results are derived from a GPR model, trained on 120 (**A**) and 199 (**B**) experimental observations dispersed through the 2D space.

Pulsed Electrolysis Supporting Data

To better understand how the three pulsing parameters (j_c, t_c, and t_r) affect production rates for electrolysis of AN or CN-only substrates, we conducted experiments implementing multiple pulse sequences at a constant substrate concentration of 1.0 mol L^{-1}. The resulting 3D maps in production rates towards ADN, PN and CDN vs. pulsing parameters are shown in Figure S9. Figure S10A displays 2D slices of these 3D maps at a constant j_c = 200 mA cm^{-2}, revealing the interplay between active and resting current durations. Hydrodimers ADN and CDN follow similar trends: production rates decrease at longer t_r, particularly at short t_c when overall reaction time is reduced. In contrast, dimer production is enhanced by increasing t_c until reaching the optimal fraction of faradaic current per pulse. Figure S10B presents 2D slices of the same maps at a constant t_r = 40 ms, showing the relationship between length and height of active pulse. Likewise, hydrodimers show similar behavior: production rates increase with longer t_c and larger j_c where faradaic current is maximized. However, at longer t_c, the effect of pulsing is expected to be diminished and behavior approaches DC operation. At even larger than the tested j_c values, reactant consumption accelerates, potentially leading to depletion in the electrode surface. This effect is already observed in the emergence of greater AN hydrogenation rates towards PN at larger j_c, which was effectively suppressed at j_c < 100 mA cm^{-2}.

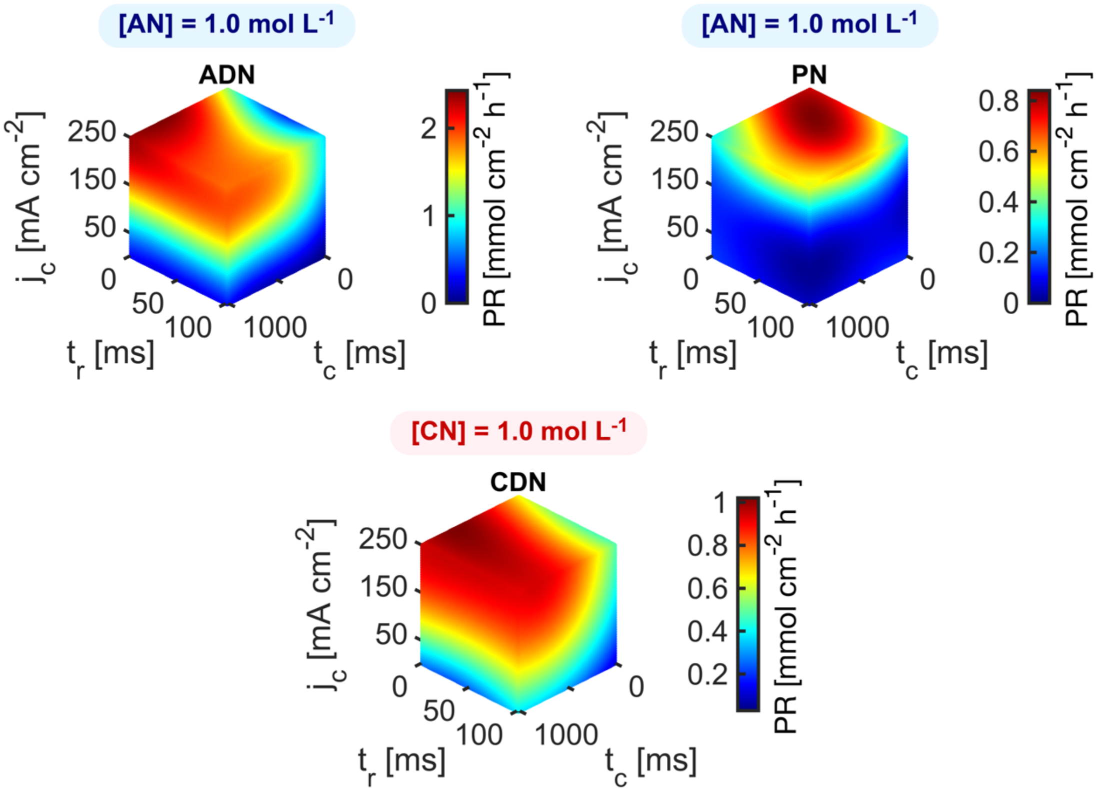

Figure S9. Pulse sequence effects on single-substrate production rates. Effect of j_c, t_r, and t_c on the production rate of ADN and PN when using AN substrate and CDN when using CN substrate. Pulsed electroreduction was carried on a Cd rod in undivided parallel reactors, at a constant cumulative cathodic charge passed of 12 C. Electrolytes contained 1.0 mol L^{-1} substrate (AN or CN), 0.5 mol L^{-1} Na_3PO_4, 0.03 mol L^{-1} EDTA, and 0.02 mol L^{-1} TBA hydroxide. The results are derived from a Gaussian process regression model, trained on 80 (AN substrate) and 77 (CN substrate) experimental observations dispersed through the 3D space.

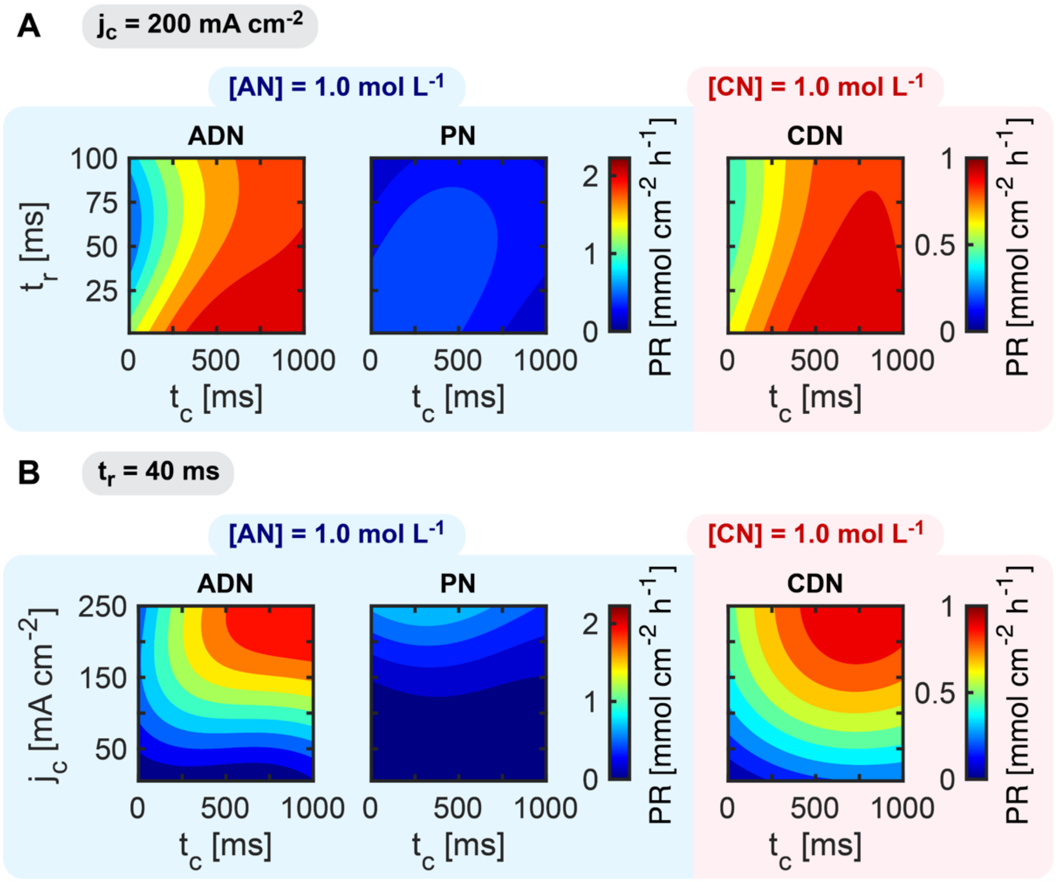

Figure S10. Pulse sequence effects on single-substrate production rates. 2D slices of the 3D surrogate models of the effect of j_c, t_r, and t_c on the production rate of ADN and PN when using AN substrate and CDN when using CN substrate, keeping constant j_c of 200 mA cm^{-2} (**A**) and constant t_r of 40 ms (**B**). Pulsed electroreduction was carried on a Cd rod in undivided parallel reactors, at a constant cumulative cathodic charge passed of 12 C. Electrolytes contained 1.0 mol L^{-1} substrate (AN or CN), 0.5 mol L^{-1} Na_3PO_4, 0.03 mol L^{-1} EDTA, and 0.02 mol L^{-1} TBA hydroxide. The results are derived from a Gaussian process regression model, trained on 80 (AN substrate) and 77 (CN substrate) experimental observations dispersed through the 3D space.

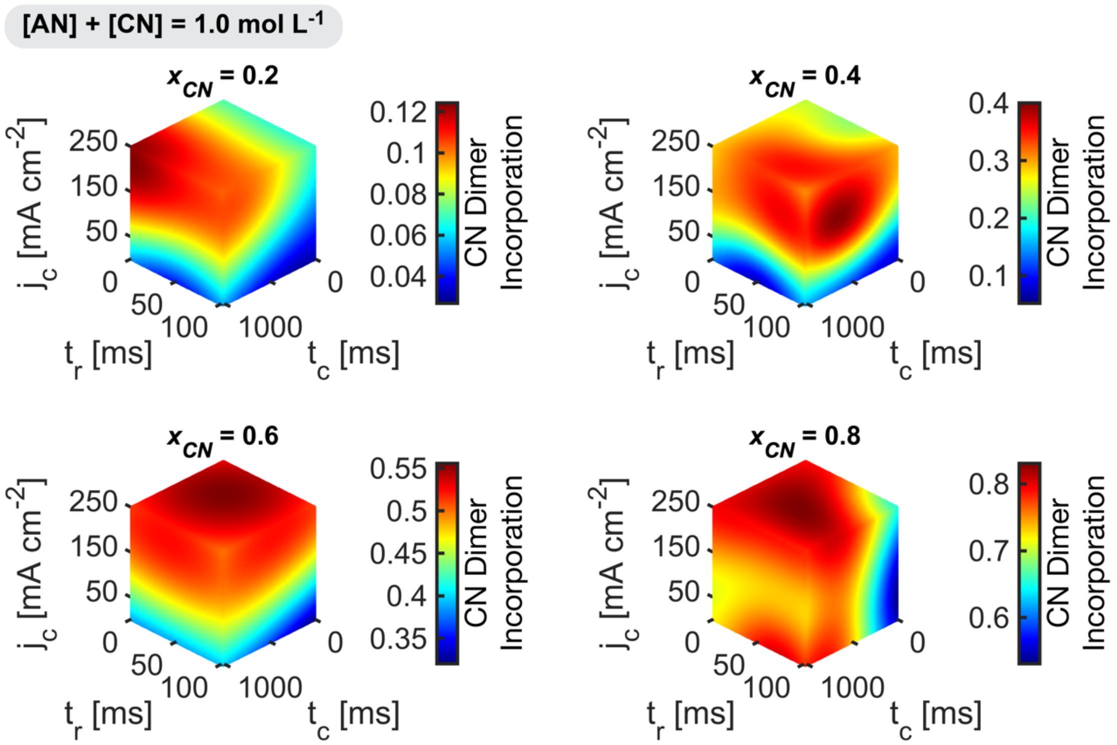

Figure S11. Tuning CN dimer incorporation through cathode pulse sequencing. Effect of j_c, t_r, and t_c on the incorporation of CN in dimers at varied CN relative molar fraction (x_{CN}). Pulsed electroreduction was carried on a Cd rod in undivided parallel reactors, at a constant cumulative cathodic charge passed of 12 C. Electrolytes contained 1.0 mol L^{-1} substrate (AN and CN combined), 0.5 mol L^{-1} Na_3PO_4, 0.03 mol L^{-1} EDTA, and 0.02 mol L^{-1} TBA hydroxide. The results are derived from a Gaussian process regression model, trained on 54 (x_{CN} = 0.2), 52 (x_{CN} = 0.4), 50 (x_{CN} = 0.6), and 55 (x_{CN} = 0.8) experimental observations dispersed through the 3D space.

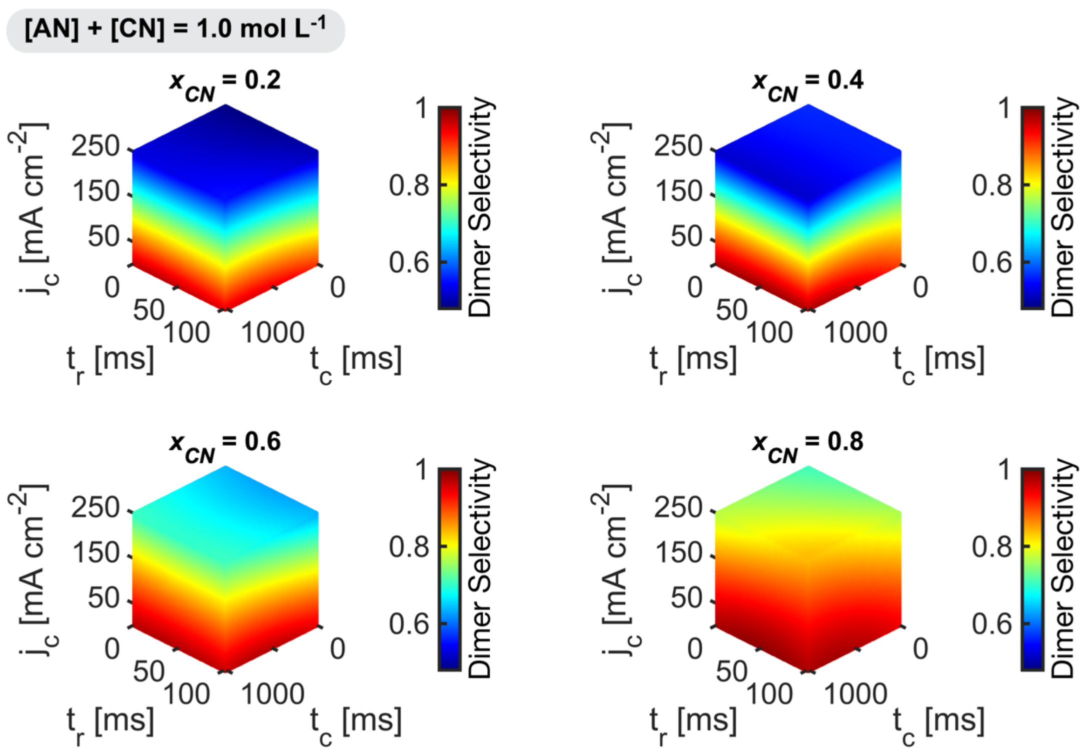

Figure S12. Tuning dimer selectivity through cathode pulse sequencing. Effect of j_c, t_r, and t_c on the dimer selectivity at varied CN relative molar fraction (x_{CN}). Pulsed electroreduction was carried on a Cd rod in undivided parallel reactors, at a constant cumulative cathodic charge passed of 12 C. Electrolytes contained 1.0 mol L^{-1} substrate (AN and CN combined), 0.5 mol L^{-1} Na_3PO_4, 0.03 mol L^{-1} EDTA, and 0.02 mol L^{-1} TBA hydroxide. The results are derived from a Gaussian process regression model, trained on 54 (x_{CN} = 0.2), 52 (x_{CN} = 0.4), 50 (x_{CN} = 0.6), and 55 (x_{CN} = 0.8) experimental observations dispersed through the 3D space.

Liquid chemical quantification

Liquid products were measured using a Shimadzu gas chromatographer equipped with a mass spectrometer (GCMS-QP2010). Each product was quantified using the calibration curves shown in Figure S13.

After the moles of products were determined based on the techniques outlined above, the Faradaic efficiency (FE) was calculated for each *i* species as:

$$FE_i = \frac{n_i F z_i}{Q} \tag{1}$$

where n_i is the number of moles of species i, F is Faraday's constant, z_i is the number of electrons transferred in the formation of a molecule of the species i, and Q is the total charge transferred. The production rate (PR) of species i was calculated using the following formula (where t_r is the total reaction time and A is the electrode surface area):

$$PR_i = \frac{n_i}{t_r A} \tag{2}$$

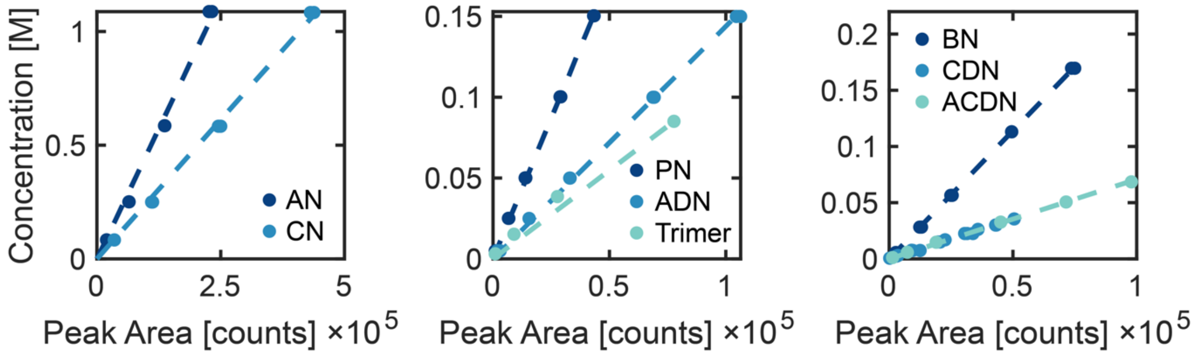

Figure S13. Gas Chromatography (GC) calibration curves for reactants and products. Relating GC total peak area to aqueous concentration. Each point represents an average of 2 samples.

The CN used in this study was obtained as a commercial mixture of cis and trans geometric isomers, which represents the standard form supplied by major chemical vendors. To verify that both isomers exhibit similar electrochemical behavior, we conducted control experiments using CN electroreduction on Cd foil at 150 mA cm^{-2} for 7 minutes in the H-cell configuration shown in Figure S4. The bulk CN concentration was maintained at 0.25 mol L^{-1} to ensure a single-phase electrolyte and prevent CN evaporation. Gas chromatography analysis of the reaction mixture before and after electroreduction (5 replicates) revealed that the conversion of cis and trans CN isomers was 43.7% and 44.5%, respectively (Figure S14). These results demonstrate that both geometric isomers exhibit nearly equivalent reactivity under the electrochemical reduction conditions employed in this work.

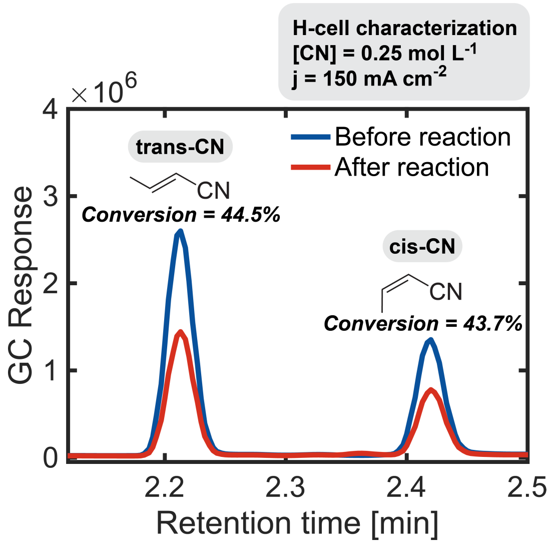

Figure S14. Gas chromatography analysis of cis/trans crotononitrile isomer conversion. Representative chromatograms showing the composition of the CN isomer mixture before and after electroreduction on Cd foil at 150 mA cm^{-2} for 7 minutes. Electroreduction was carried out in a divided H-cell reactor, and the electrolyte contained 0.25 mol L^{-1} CN, 0.5 mol L^{-1} Na_3PO_4, 0.03 mol L^{-1} EDTA, and 0.02 mol L^{-1} TBA hydroxide.

Standard solutions of CDN and ACDN were prepared by purification of organic product mixtures via liquid-liquid extraction with dichloromethane and rotary evaporation. The purified products were dissolved in toluene, quantified using a Bruker Avance III 400 NMR Spectrometer and with ethylene carbonate (EC) as an internal standard. The moles of molecule *x* were calculated using the following formula:

$$n_x = \frac{I_x}{I_{EC}} \cdot \frac{H_{EC}}{H_x} \cdot n_{EC} \quad (3)$$

where *n*, *I*, and *H* are the moles, integral area, and number of nuclei of the compound of interest (*x*) and the calibrant (*EC*), respectively.

Figure S15A shows a sample spectrum of CDN in D_2O. CDN, with two chiral centers, produces three stereoisomers: (R,R), (S,S), and (R,S), where (R,S) is identical to (S,R) due to symmetry. In

the NMR spectrum of the mixture, each peak appears as two signals: one corresponding to the (R,S) diastereomer and the other to the overlapping (R,R) and (S,S) enantiomers.[6] This behavior is expected, as diastereomers differ in their chemical environments and thus exhibit distinct shifts, while enantiomers, being mirror images, are indistinguishable in an achiral solvent.[7] All stereoisomers of CDN overlap in the same region in the GC, and their peaks are integrated together to quantify the combined concentration as a single response. In contrast, ACDN, shown in Figure S15B and dissolved in toluene-d9, contains a single chiral carbon and displays each peak as a single signal in the NMR spectrum when analyzed in an achiral solvent, as the R and S enantiomers are chemically indistinguishable under these conditions.[8]

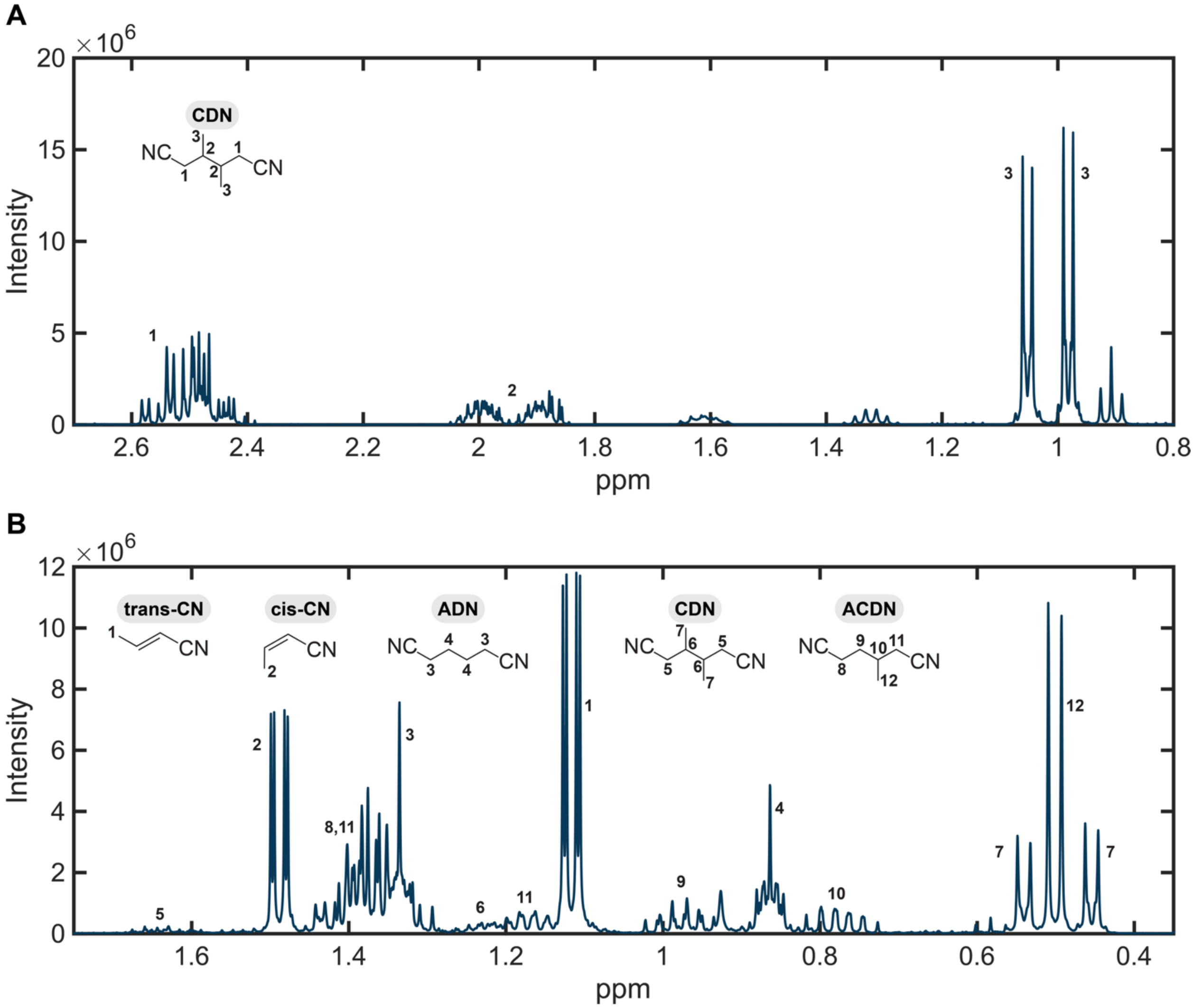

Figure S15. Sample H^{1}NMR spectra of relevant molecules to this study. Obtained in a 400 MHz NMR: 3,4-dimethyladiponitrile (CDN), crotononitrile (CN), adiponitrile (ADN), and 3-methyladiponitrile (ACDN).

References

1. Wong, T.-T.; Luk, W.-S.; Heng, P.-A., Sampling with Hammersley and Halton Points. *Journal of Graphics Tools* **1997,** *2* (2), 9-24.
2. Blanco, D. E.; Dookhith, A. Z.; Modestino, M. A., Enhancing selectivity and efficiency in the electrochemical synthesis of adiponitrile. *Reaction Chemistry & Engineering* **2019,** *4* (1), 8-16.
3. Wu, S.; Zhang, H.; Huang, X.; Liao, Q.; Wei, Z., Acrylonitrile Conversion on Metal Cathodes: How Surface Adsorption Determines the Reduction Pathways. *Industrial & Engineering Chemistry Research* **2021,** *60* (23), 8324-8330.
4. Mathison, R.; Atwi, R.; McConnell, H. B.; Ochoa, E.; Rani, E.; Akashige, T.; Rohr, J. A.; Taylor, A. D.; Avalos, C. E.; Aydil, E. S.; Rajput, N. N.; Modestino, M. A., Molecular Processes That Control Organic Electrosynthesis in Near-Electrode Microenvironments. *J Am Chem Soc* **2025,** *147* (5), 4296-4307.
5. Bloomquist, C. K.; Dogan, M.; Harris, J. S.; Herzog, B. D.; Tenn III, W. J.; Aydil, E. S.; Modestino, M. A., Understanding the effects of forced and bubble-induced convection in transport-limited organic electrosynthesis. *Reaction Chemistry & Engineering* **2024,** *9* (4), 930-939.
6. Lin, J.; Tsang, C.; Lieu, R.; Zhang, K., Method screening strategies of stereoisomers of compounds with multiple chiral centers and a single chiral center. *J Chromatogr A* **2020,** *1624*, 461244.
7. Testa, B., Organic stereochemistry. Part 2: Stereoisomerism resulting from one or several stereogenic centers. *Helvetica Chimica Acta* **2013,** *96* (2), 159-188.
8. Huang, A.; Zhang, L.; Li, D.; Liu, Y.; Yan, H.; Li, W., Asymmetric One-Pot Construction of Three Stereogenic Elements: Chiral Carbon Center, Stereoisomeric Alkenes, and Chirality of Axial Styrenes. *Org Lett* **2019,** *21* (1), 95-99.
